# Unveiling Glucosinolate Diversity in *Brassica* Germplasm and In Silico Analysis for Determining Optimal Antioxidant Potential

**DOI:** 10.3390/antiox13030376

**Published:** 2024-03-19

**Authors:** Kanivalan Iwar, Kebede Taye Desta, Kingsley Ochar, Seong-Hoon Kim

**Affiliations:** 1 National Agrobiodiversity Center, National Institute of Agricultural Science, Rural Development Administration, Jeonju 5487, Republic of Korea; kani_valan@yahoo.in (K.I.); kingochar@yahoo.com (K.O.); 2 Department of Botany, Bharathiar University, Coimbatore 641046, Tamil Nadu, India; 3 Department of Applied Chemistry, Adama Science and Technology University, Adama 1888, Ethiopia; 4 Council for Scientific and Industrial Research, Plant Genetic Resources Research Institute, Bunso P.O. Box 7, Ghana

**Keywords:** *Brassica*, glucosinolates, in silico analysis, molecular docking analysis

## Abstract

This study explored the glucosinolate (GSL) content in *Brassica* plants and utilized in silico analysis approach to assess their antioxidant capabilities. GSLs, present abundantly in *Brassica* vegetables, offer potential health advantages, including antioxidant effects. Employing Ultra-Performance Liquid Chromatography (UPLC) coupled with tandem mass spectrometry (MS/MS), major GSLs were identified in 89 accessions from diverse species and subspecies. Statistical analysis and principal component analysis unveiled significant GSL variation and potential correlations among the *Brassica* germplasms. This study unveils the dominance of aliphatic GSLs over aromatic and indolyl compounds in all the accessions. Notably, Gluconapin (GNA) (33,049.23 µmol·kg^−1^ DW), Glucobrassicanapin (GBN) (9803.82 µmol·kg^−1^ DW), Progoitrin (PRO) (12,780.48 µmol·kg^−1^ DW) and Sinigrin (SIN) (14,872.93 µmol·kg^−1^ DW) were the most abundant compounds across the analyzed accessions. Moreover, in silico docking studies predicted promising antioxidant activity by evaluating the interactions of each GSL with antioxidant enzymes. Specifically, Sinigrin and Gluconapin exhibited a notably weaker influence on antioxidant enzymes. This provides key insights into the antioxidant potential of *Brassica* germplasm and highlights the importance of in silico analysis for evaluating bioactive properties. In general, the results of this study could be utilized in breeding programs to maximize GSL levels and antioxidant properties in *Brassica* crops and for developing functional foods with enhanced health benefits.

## 1. Introduction

The Brassicaceae family, characterized by its monophyletic nature and predominantly hermaphroditic species, encompasses approximately 346 genera and 4202 species globally, primarily found in temperate regions such as the Irano-Turanian Region, the Mediterranean, and North Western America [1]. Notable members of this family include various vegetables and flavoring plants with significant economic importance, such as *Brassica oleracea* cultivars (e.g., broccoli, cabbage, and cauliflower), *Armoracia rusticana* (horseradish), *B. nigra* (mustard), *B. napus* (canola), *B. rapa* (turnip), *Eutrema japonicum* (wasabi), and *Raphanus sativus* (radish), among others. Additionally, several ornamental species and model organisms used for molecular research, like *Arabidopsis thaliana*, contribute to the diverse representation within the Brassicaceae family. Taxonomic studies on *Brassica species* date back to the 18th century, with notable contributions from outstanding researchers such as Tournefort [2], Linnaeus [3], De Candolle [4], Bentham and Hooker [5], Baillon [6], Prantl [7], Schulz [8], Beilstein et al. [9], and Branca and Cartea [10]. The genetic boundaries of *Brassica* crops’ gene pools, categorized into primary, secondary, and tertiary gene pools, regulate the genetic resources utilized in breeding programs [11]. Particularly, *Brassica* vegetables are agriculturally significant due to their rich glucosinolate (GSL) content, which contributes to their nutritional value [12].

GSLs, major bioactive compounds found in Brassicaceae members, are amino acid derivatives known for their antioxidant and cancer-protective activities [13,14]. GSLs serve as a defense mechanism against tissue disruption and herbivory, with their hydrolyzed products acting as inactive biological responses [15]. Recent studies have highlighted the regulatory functions of GSLs in inflammation, phase I metabolism, stress response, and antioxidant and antimicrobial properties, further emphasizing their therapeutic potential [16]. Structurally, GSLs are classified based on their precursor amino acids and the types of degradation products they yield, including isothiocyanates (ITC), oxazolidine-2-thione, and non-volatile/volatile compounds [17]. The major GSL groups include aliphatic, aromatic, and indole, derived from specific amino acids such as alanine, phenylalanine, tyrosine, and tryptophan, respectively [17].

*Brassica* wild relatives serve as a rich genetic resource harboring desirable alleles governing quantitative traits of economic significance, including those related to nutrition, therapeutic applications, and biocidal properties [18]. The primary gene pool, centered around *B. oleracea*, has been extensively studied, and investigations into various gene pools and their potential utility have been conducted [10]. *Brassica* species and their relatives exhibit diverse morphological characteristics crucial for their growth and development. With approximately 42 species and 47 subspecies recognized taxonomically [1], it is noteworthy that *B. macrocarpa* (Guss.) is critically endangered, while *B. hilarionis* Post and *B. villosa* subsp. *drepanensis* (Caruel) are endangered. On the other hand, *B. repanda* subsp. *glabrescens* (Poldini) is considered a vulnerable species, while *B. rupestris* (Raf.) and *B. villosa* (Biv.) are near-threatened. *B. balearica* Pers., *B. barrelieri* (L.) Jank, *B. cretica* Lam., *B. elongata* Ehrh., *B. montana* Pourr., and *B. nivalis* Boiss. & Heldr. were categorized as being of least concern, while species for which data are insufficient include *B. cadmea* Heldr. *ex* O.E.Schulz, *B. incana* Ten., and *B. oleracea* L. (Wild). Overall, these 15 species are categorized as threatened according to the IUCN red list [19]. Conservation efforts encompass both in situ and ex situ activities aimed at preserving the wide genetic diversity within the *Brassica* genus [19]. Germplasm resource data for *Brassica*, retrieved from GENESYS [20], GRIN Global [21], European Co-operative Programme for Plant Genetic Resources [22], and FAOSTAT [23], further contribute to the comprehensive understanding and conservation of *Brassica* genetic resources.

Computational analysis plays a crucial role in biochemistry by providing insights into the structure and functions of biomolecules. This study offers a pioneering in silico exploration of three-dimensional structure, active site machinery, and enzyme–substrate interactions, marking a significant advancement in the field of biochemistry [24,25,26,27,28]. It also underscores the analytical and predictive capabilities of computational analysis, particularly in elucidating the intricate relationship between the structure and functions of biomolecules through enzyme–substrate docking studies. Recent developments in computational strategies, particularly through in silico techniques, have markedly improved the drug discovery landscape. These approaches are notably efficient in analyzing the intricate poly-pharmacological profiles of the phytochemicals found, providing a solid framework for pinpointing potential therapeutic agents [24,26,27,28]. The interaction between proteins and ligands is often described using the lock-and-key paradigm, where the protein serves as the lock and the ligand acts as the key. This analogy underscores the specificity required for a ligand to bind effectively to its target. Through the process of molecular docking, a vast array of phytochemicals can be virtually screened against a biological target. This involves calculating the likelihood of a ligand binding to a target using sophisticated scoring algorithms, thereby facilitating the identification of promising lead compounds [29]. Utilizing in silico molecular docking and computational molecular modeling, researchers can explore the potential anti-inflammatory, antioxidant, and antidiabetic properties of bioactive compounds, paving the way for the development of new therapeutic agents [30]. 

The analysis of GSLs’ content and their biological effects is crucial due to the variation patterns observed in their distribution and impact on biological systems. Recent studies have emphasized the importance of understanding these patterns for both agricultural and health-related applications. For instance, the varying profile patterns in GSL content can significantly influence the nutritional quality and pest resistance of crops [31]. Furthermore, the biological effects of GSLs, including their roles as antioxidant, anti-inflammatory, and cancer prevention agents, are highly dependent on their specific content and composition, which exhibit variations among different plant species and even within different parts of the same plant [32]. Moreover, the relationship between GSL content and bioactivity underscores the complexity of their mechanisms of action. GSLs may exhibit increased bioactivity only beyond specific thresholds, which can vary depending on the environmental conditions and genetic factors influencing a plant’s metabolism [33]. The non-linear patterns in GSL content and biological effects highlight the necessity for advanced analytical techniques and interdisciplinary approaches to fully understand and harness the potential of GSLs. Such studies pave the way for developing targeted strategies for crop improvement and for the formulation of GSL-based health supplements with optimized efficacy [31,32]. Thus, this study aims to analyze GSL profiles and characterize the variations in their content in diverse *Brassica* germplasms. We examined the correlation between GSL diversity and the prediction of GSLs’ influence on antioxidant enzymes through in silico screening utilizing a computational method. In silico screening of potent antioxidant metabolites is crucial for drug development and enhancing crop improvement by using high GSL content for potential health benefits.

## 2. Materials and Methods

### 2.1. Chemical Reagents

All the chemicals used in this study were of analytical grade and obtained from Sigma-Aldrich (St. Louis, MO, USA) and ThermoFisher Scientific Korea (Seoul, Republic of Korea). Six of the seventeen glucosinolate standards, namely, Progoitrin (PRO), Epiprogoitrin (EPI), Glucobrassicanapin (GBN), Glucoiberin (GIB), Glucoraphenin (GRE), and Sinalbin (SNB), were sourced from Phytolab (Martin Bauer, KG, Vestenbergsgreuth, Germany), while the remaining eleven GSLs were purchased from Phytoplan (Neuenheimer, Heidelberg, Germany).

### 2.2. Collection and Cultivation of Plant Materials

In this study, 89 *Brassica* germplasms preserved at the National Agrobiodiversity Center (RDA-Genebank) in the Republic of Korea were used as the studied plant materials. The selected genetic materials were distributed across different *Brassica* subspecies, including *B. oleracea* var. *medullosa* Thell., *B. rapa* subsp. *campestris* (L.) A.R.Clapham, *B. rapa* subsp. *narinosa* (L.H.Bailey) Hanelt, *B. rapa* subsp. *nipposinica* (L.H.Bailey) Kitam., *B. rapa* subsp. *pekinensis* (Lour.) Kitam., *B. rapa* subsp. *rapa* L., and unidentified *Brassica* sp., with cultivars, landraces, and wild relatives. The list of germplasms used in these experiments is provided in Table S1. *Brassica* is an outcrossing crop, and measures were taken to minimize cross-fertilization. A mesh material smaller than the size of the pollen holes was employed during the second generation (2021–2022) in the greenhouse from February to June to reduce pollen scattering. Furthermore, the multiplied seeds were cultivated in the field from September to November, and heterogeneous germplasms were systematically separated and continuously removed based on phenotype to ensure purity maintenance.

### 2.3. Sample Preparation: Pre-Treatment and Extraction

Leaves were randomly harvested from each plant accession and immediately placed into polyvinyl bags. Subsequently, the leaves underwent lyophilization using an LP500 vacuum freeze drier from Ilshinbiobase Co. (Dongducheon, Republic of Korea) for 2 days (48 h). Following lyophilization, the dried leaves were ground into a fine powder. The powdered leaves were then returned to −80 °C storage until further processing. The extraction of GSLs from the powdered leaves followed a method previously established by Kim et al. [34]. Specifically, 0.1 g of harvested leaves was combined with 5 mL of 80% methanol and incubated at 25 °C for 30 min. The mixture was then continuously shaken at 120 rpm for an additional 30 min at 25 °C. Subsequently, the mixture was centrifuged at 14,000 rpm for 10 min at 4 °C, and the resulting supernatants were carefully transferred into clean vials for further analysis.

### 2.4. Identification of GSLs Using UPLC-MS/MS

The analysis of GSLs was conducted, using three replicates, via the Acquity Ultra-Performance Liquid Chromatography system (Waters, Milford, CT, USA), coupled with the Xevo™ TQ-S system developed by MS Technologies (UK), according to the method described by Kim et al. [34]. Specifically, 5 µL of sample extract was injected and separated using BEH C18 column (1.7 µm, 2.1 × 100 mm) (Waters, MS Technologies, Wilmslow, UK) set at a temperature of 35 °C for elution. Here, 0.1% trifluoroacetic acid in water served as eluent A, while eluent B consisted of 0.1% trifluoroacetic acid in methanol. The elution was performed at a flow rate of 0.5 mL/min. The elution conditions were programmed as follows: 100% of A from 0.0 to 1.0 min and maintenance at 100% of A from 1.0 to 7.0 min, followed by a gradient of 100–80% of A from 7.0 to 10 min, 80–0% of A from 10 to 11 min, and 0–100% of A from 11 to 15 min, and finally maintenance at 100% of A 10 for 11 min. Multiple-reaction monitoring (MRM) in negative electrospray ionization mode was used for the detection and quantification of the GSLs [35]. The MS/MS parameters included capillary and cone voltages set at 3 kV and 54 V, respectively, for ionization. Identification of GSLs was accomplished by comparing their retention times and MS/MS fragmentation spectra with those of commercially available standards. Method validation included assessing precision and accuracy through linear, intra-day, and inter-day precision measurements. Calibration curves were constructed using stock solutions (1 mg mL^−1^) prepared by dissolving 10 mg of individual GSLs in methanol. GSL concentrations were determined based on these calibration curves and expressed as µmol GSLs kg^−1^ sample dry weight (DW). Fresh batches of test solutions were consistently prepared before sample analysis.

### 2.5. Multivariate Analysis

This study utilized Microsoft Excel 2021 MSO (Ver. 2401) for numerical data sampling, Origin Pro v2024 for Principal Component Analysis, SPSS 16.0 for Pearson coefficient analysis, and the SR Plots online web server [36] for heat dendrogram and hierarchical cluster analysis. Employing hierarchical clustering, PCA, and Pearson’s correlation analysis enabled an exploration of variable relationships in the diversity analysis of 10 GSL profile values from 89 *Brassica* accessions. This approach provided nuanced insights, facilitating informed decision making based on complex datasets.

### 2.6. In Silico Screening and Molecular Docking Analysis

This methodology involves identifying potential drug candidates with favorable pharmacokinetic properties and screening for antioxidant potential compounds through computational modeling techniques. Firstly, ligands’ SMILES were retrieved from PubChem [37] using the CIDs Glucobrassicanapin (5485207), Gluconapin (9548620), Gluconasturtiin (656555), Glucotropaeolin (9548605), and Sinigrin (6911854) as SDF 3D conformer files. The pharmacokinetic parameters of the ligands, including molecular weight (MW), molar refractivity (MR), solubility (S), and bioavailability, were predicted using the SwissADME online tool [38]. This tool also provided information on absorption in the human gastrointestinal tract (HIA) and brain penetration properties. Furthermore, the selected GSLs were used to predict biologically potent activity using the Way2Drug predictive web server [39] in order to understand the relevant biochemical interactions. The protein targets for evaluation of antioxidant activity were scrutinized based on the active pathways involved and previous work. Catalase (CAT), Glutathione peroxidase (GPX), and Superoxide dismutase (SOD) were selected through the simulation of activity, wherein the protagonists increase the activity of CAT, GPX, and SOD and may overcome the ROS induction related to stress and bioactive compounds that act as agonists [40]. CAT, GPX, and SOD were selected as targets for the antioxidant activity of phytochemicals. From the protein data bank (PDB), we retrieved the targeted 3D protein FASTA sequences, with PDB IDs of 7VD9 (CAT), 2P31 (GPX), and 7KKU (SOD) [41], and homology model targets were structured and assessed using the SWISS-MODEL online workspace [42,43,44]. The remodelled structures of targets were used to predict the ligand binding sites using the PrankWeb [45]. Molecular docking analysis was conducted using the Webina ideal library web tool [46] that runs the AutoDock Vina process and calculates affinity and root mean square deviation (RMSD) score. The results were visualized using the Pymol 2.5 visualization tool, and the interactions between the bioactive compounds and protein targets were analyzed.

## 3. Results and Discussion

In this study, a total of 89 *Brassica* germplasm collections encompassing various species and subspecies were analyzed for their major GSL compositions. The subspecies and varieties are synonymous with *B. rapa* L. and *B. oleracea* L., respectively [1]. We identified and quantified ten GSLs, including Glucobrassicanapin (GBN), Glucoberteroin (GBE), Glucoerucin (GER), Gluconapin (GNA), Progoitrin (PRO), and Sinigrin (SIN) from the aliphatic group and Glucobarbarin (GBB), Glucotropaeolin (GTL), Gluconasturtiin (GNS), and Glucobrassicin (GBS) from the aromatic and indolyl groups. Our analysis revealed substantial variability in GSL composition among the 89 *Brassica* collections. The content of GBB, GBE, GBN, GBS, GER, GNA, GNS, GTL, PRO, and SIN in the entire population ranged from 0 to 150.694, 0 to 3217.82, 0.035 to 9803.82, 28.07 to 2098.26, 0.15 to 2903.38, 0.21 to 33,049.23, 3.47 to 1494.47, 0.30 to 40.77, 1.86 to 12,780.48, and 0.04 to 14,872.93 µmol·kg^−1^ DW, respectively. We observed a predominance of aliphatic GSLs over aromatic and indolyl compounds. Notably, Gluconapin (GNA) (33,049.23 µmol·kg^−1^ DW), Glucobrassicanapin (GBN) (9803.82 µmol·kg^−1^ DW), Progoitrin (PRO) (12,780.48 µmol·kg−1 DW), and Sinigrin (SIN) (14,872.93 µmol·kg^−1^ DW) emerged as abundant compounds across the analyzed accessions, a result consistent with the established standards presented in Tables S2 and S3.

*Brassica* vegetables GSL profiles have high variation in both parts and plants [47]. The composition of GSLs in various *Brassica* species is significantly influenced by environmental factors such as climate and farming practices, including soil quality, fertilization methods, harvest timing, and plant part utilization [48]. 

In particular, in this study, aliphatic metabolites such as GBE and GBB were either detected at minimal levels or not detected at all. Similarly, GBN, GNA, and SIN also exhibited very low concentrations overall. This analysis highlighted significant variation in glucosinolate composition among *Brassica* species. This variation in environmental conditions results in distribution differences among taxa, from families to varieties, with an emphasis on possessing one to three principal compounds [49]. The present findings align with previous work on *Brassica rapa* varieties revealing GSL variation in the leaves of broccoli, Chinese cabbage, Pak choi, rapeseed, Sarson, and turnip [50,51]. Lee et al. [52] observed wide variability in glucosinolate content in *B. rapa* L. ssp. *pekinensis* varieties, with significant concentrations of various aliphatic and indolyl GSLs. Moreover, Maldonade et al. [53] unveiled the impact of soil mineral fertilization on Brazilian collard varieties, resulting in significant variations in GSL content. These findings underscore high-throughput analysis’s efficacy in identifying and quantifying GSLs with varying ranges of concentration.

### 3.1. Brassica Accessions: GSL Variation According to Origin

This analysis elucidates the diverse distribution of GSL compounds in *Brassica* accessions, revealing distinct abundance patterns across various genetic backgrounds. It provides crucial insights into the variability and distribution of GSLs in *Brassica* species. GNA occurs as a prominent and abundant compound. Notably, *B. rapa* subsp. *nipposinica* Taiwan Landraces (100406) exhibited the highest levels of GNA (33,049.23 µmol·kg^−1^ DW) across all the studied samples, while the Spain landrace of *Brassica* sp. (K018856) showed the lowest GNA concentration, namely, 0.21 µmol·kg^−1^ DW. GBN was identified as a prevalent GSL compound across all samples. Particularly, *B. rapa* subsp. *peikensis* Taiwan Landrace (100353) accessions exhibited the highest levels of GBN (9803.82 µmol·kg^−1^ DW). Conversely, the lowest GBN concentrations were recorded in the landraces of *Brassica* sp. (K018853) (0.19 µmol·kg^−1^ DW). PRO emerged as the third-most-abundant GSL present in the analyzed accessions. Remarkably, the Peru cultivar of *Brassica* sp. (K229558) exhibited the highest PRO concentrations (12,780.48 µmol·kg^−1^ DW). The lowest concentration of PRO (1.86 µmol·kg^−1^ DW) was recorded in Spain landraces of *Brassica* sp. (K018856). SIN was notably abundant in the Costa Rica landrace of *B. rapa* subsp. *peikensis* (K043728) (14,872.93 µmol·kg^−1^ DW). Conversely, its presence was minimal in the South Korean landrace of *B. rapa* subsp. *rapa* (K255223) (0.047 µmol·kg^−1^ DW). The presence of GTL in the studied accessions was notably lower compared to that in other GSLs. The Taiwan landrace of *B. rapa* subsp. *peikensis* (100356) exhibited the highest recorded content, namely, 40.77 µmol·kg^−1^ DW, while it was the least detected in Spain landraces of *Brassica* sp. (K018856). GER exhibited high levels in the Georgia landrace of *B. rapa* subsp. *rapa* (K257600) (2903.38 µmol·kg^−1^ DW). The lowest GER content (0.15 µmol·kg^−1^ DW) was exhibited in the cultivar of *Brassica* sp. (216480). GNS content was found to be abundant in *B. rapa* subsp. *peikensis* cultivar from China (K037469), with 1494.47 µmol·kg^−1^ DW, and minimal in the Spain landrace of *Brassica* sp. (K018856), with 3.47 µmol·kg^−1^ DW. GBE exhibited significant levels in the *B. rapa* subsp. *narinosa* landrace (228170) from Taiwan (3217.82 µmol·kg^−1^ DW), while it was undetectable in the *Brassica* sp. cultivars and landrace accessions (216480, K004273, K018853, and K018856). On the other hand, Glucobarbarin (GBB) was identified as the least-recorded glucosinolate in all the studied *Brassica* accessions. While it was detected in relatively high amounts in the *B. rapa* subsp. *peikensis* landrace (100354) from Taiwan (150.69 µmol·kg^−1^ DW), it was absent in the Ugandan cultivars of *B. oleracea* var. *medullosa* (339591) and in *B. rapa* subsp. *peikensis* (K022386, K193859) from China and South Korea. Similarly, among *B. rapa* subsp. *campestris* of the Italian wild relative (K000507), *B. rapa* subsp. *rapa* of Mongolia landrace (K002855) from China and South Korea, and *B. rapa* subsp. *rapa* of the Mongolia landrace. GBS content was found to be the highest observed in the *B. rapa* subsp. *narinosa* landrace (306666) from Japan (2098.26 µmol·kg^−1^ DW), whereas its presence was minimal in the *B. rapa* subsp. *rapa* landrace (907305) from Russia (28.07 µmol·kg^−1^ DW) (Table S2).

**Figure 1:**
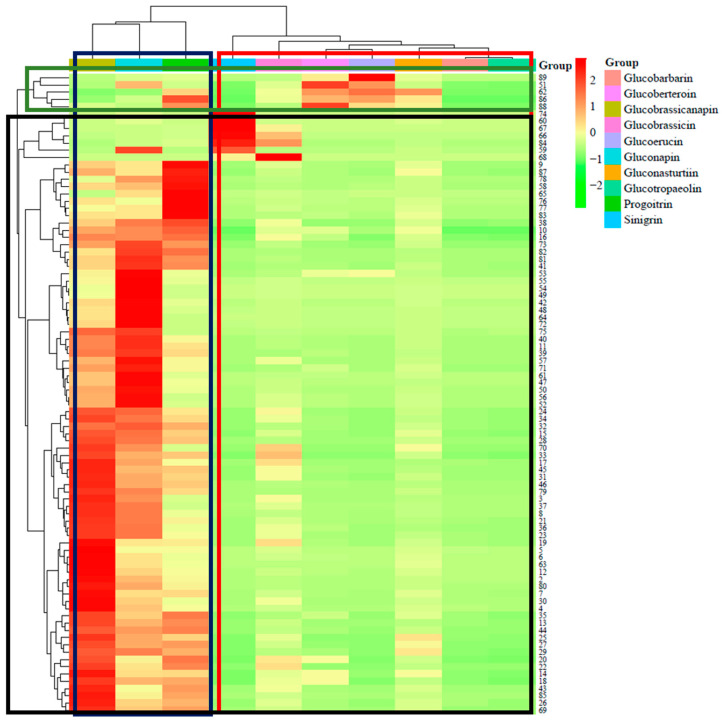
Cluster heat map of GSL profiles in *Brassica* germplasms. Shown horizontally and highlighted in green are clusters comprising *B. oleracea* var. *medullosa* along with diverse cultivars and landraces of *Brassica* species. The black-highlighted clusters represent various subspecies of *B. rapa* with *Brassica* species landraces and cultivars from different regions. Shown vertically and highlighted in red are Glucobrassicanapin, Gluconapin, and Progoitrin, while highlighted in red are Sinigrin, Glucobrassicin, Gluconasturtiin, Glucoberteroin, Glucoerucin, Glucobarbarin, and Glucotropaeolin.

The clustering analysis revealed distinct patterns in the distribution of GSL content among various *Brassica* species and accessions. Notably, GNA, GBN, and PRO clustered together, suggesting their high abundance and potential interrelatedness. Conversely, SIN, GBS, GNS, GER, GBE, GBB, and GTL formed a separate cluster, with SIN exhibiting a notable relativeness with high abundance, particularly in association with the very-low-value spots attributed to *B. rapa* subsp. *rapa*. Despite the separation observed in the heatmap, both *Brassica* species and GSLs clustered together, indicating similarities in GSL content among *Brassica* species despite differences in their overall profiles. This finding suggests the potential utility of GSL content as a marker for classifying unidentified species based on known accession species. Moreover, the clustering analysis grouped *Brassica* species into two distinct groups, with one group predominantly consisting of *B. rapa* L., encompassing multiple subspecies and unidentified species. This observation underscores the dominance and prevalence of *B. rapa* L. within the studied *Brassica* accessions, highlighting its significance in GSL content variation (Figure 1).

Previous studies showed that the distribution of GSLs varies across different plant parts in terms of concentration and profile, with 3–4 predominant GSLs typically being observed, although up to 15 varying GSLs can be detected in a single plant [50,54]. Notably, reproductive parts such as seeds and siliques tend to possess higher concentrations of GSLs compared to young greens and old leaves, and most sprouts exhibit a richer GSL profile compared to mature plants [55]. Similarly, *Brassica juncea* showcases a variety of bioactive GSL metabolites, including GBS, GNA, GTL, PRO, and SIN, distributed throughout the plant [56]. Recent studies have also consistently emphasized that the Korean rapeseed and canola cultivars have significant PRO content, which aligns with the findings of Park et al. [57] and Kim et al. [58]. Argentieri et al. [59] mentioned that GBS was a predominant GSL in the *B. oleracea* mugnolo variety, and white cabbage root cultivars were found to have GNS in substantial concentrations, underscoring its potency as a robust GSL [60]. Likewise, GER, prominent in *Raphanus sativus* cultivars along with glucoraphanin, plays a significant role in health and providing dietary benefits [61]. Kwon et al. [62] reported SIN variations in *B. juncea* cultivars. *Brassica* vegetables, including Indian cold arid leafy selections like *Lepidium latifolium* L., were noted to be SIN-rich sources according to Kaur et al. [63]. Zaman et al. [64] explored the genetic diversity in SIN content in *Nasturtium officinale* L. Furthermore, Torras-Claveria et al. [65] utilized *K*-means clustering to differentiate *Narcissus* ornamental varieties based on their alkaloid profiles, leading to the segregation of varieties into distinct clusters, and Essoh et al. [66] identified distinct clusters of *Brassica* species separate from *Diplotaxis* and *Erucastrum* species, emphasizing specific compositions of *Brassica* species with aliphatic compounds through a comparative analysis of GSL profiles.

### 3.2. Principal Component Analysis (PCA)

**Figure 2:**
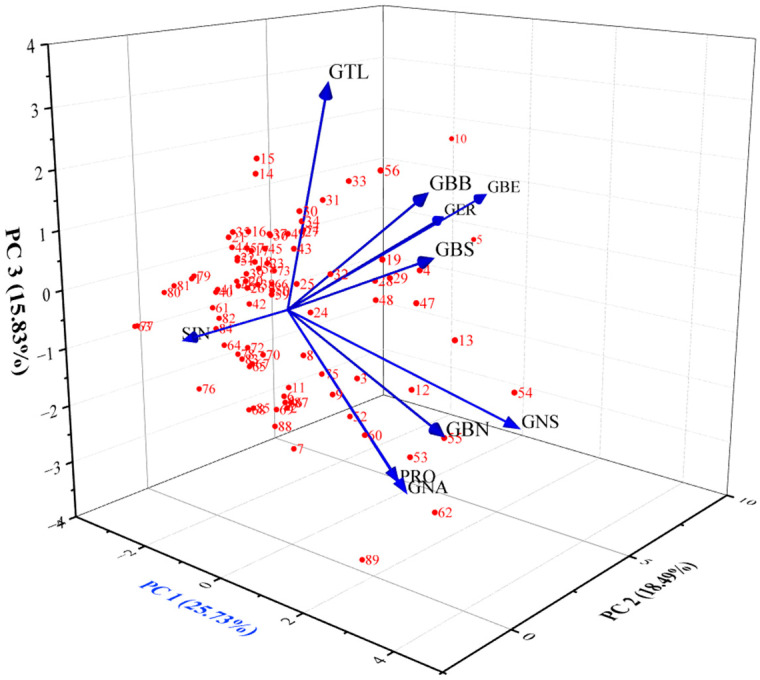
Principal Component Analysis of glucosinolate composition in *Brassica* germplasm.

In this study, PCA was utilized to analyze the chemical composition of various GSLs, yielding insightful results regarding their structural diversity and contribution to overall variance. The coefficients of the principal components (PCs) and corresponding eigenvalues provide valuable information about the underlying patterns within the GSLs. The PCA provided four principal components (PC1-PC4) derived from (PCA) GSL profiles. These coefficients offer valuable insights into the contribution of individual GSLs to the overall chemical diversity captured by each principal component. In PC1, GSLs such as GBB, GBN, GBS, and GNS exhibited positive coefficients, indicating a positive correlation with this principal component. Conversely, GSLs like GER and SIN displayed negative coefficients, suggesting a negative correlation with PC1. This suggests that PC1 likely represents broad variation among the GSLs, with some contributing positively and others contributing negatively. In PC2, GSLs such as GBE, GER, and GNA show higher positive coefficients, indicating a stronger positive correlation with PC2. Conversely, GBB and SIN have negative coefficients, suggesting a negative correlation with PC2. PC2 likely captures a different aspect of chemical diversity compared to PC1. In PC3 and PC4, there were similar patterns of positive and negative coefficients, albeit with different sets of GSLs. This indicates that PC3 and PC4 likely capture additional nuances in the chemical profiles of the GSLs, contributing to the overall variance explained by the PCA. The magnitude of eigenvalues reflects the amount of variance explained by each principal component. Higher eigenvalues indicate principal components that capture more significant proportions of the total variance in a dataset. For instance, SIN accounts for 25.73% of the total variance, indicating its substantial contribution to the overall variability in the dataset. The four principal components (SIN, GBE, GER, and GTL) together explain 70.14% of the total variance. PCA quantifies the significance of principal components in capturing variance within a GSL (Figure 2, and Table 1).

The coefficients extracted from the Principal Component Analysis (PCA) offer valuable insights into the individual contributions of GSL compounds to the observed variance in content across *Brassica* species germplasms [67]. These coefficients provide an understanding of the relative significance and directions of influence of each GSL on the identified principal components of metabolic diversity within *Brassica* species. The presence of aliphatic compounds in the PCA results is highlighted by the coefficients, with numerous aliphatic GSLs making notable contributions to the principal components [68]. These findings underscore the diverse GSL profiles among *Brassica* species and the potential for targeted breeding efforts to enhance specific GSLs for improved agronomic and nutritional traits [34,67,69]. 

### 3.3. Pearson Correlation Analysis

The *Pearson* correlation analysis of GSLs in *Brassica* species has revealed significant associations between various GSLs, indicating complex interactions within these compounds (Table 2). Specifically, the strong positive correlation between GER and GBE (r = 0.760) suggests a closely linked biosynthetic or regulatory pathway between these two GSLs. Similarly, the positive correlations observed between GBN and GNS (r = 0.518), GBB (r = 0.303), and GBS (r = 0.329) indicate that the presence or concentration of one GSL may influence the presence or concentration of others, possibly due to shared precursors or co-regulation of their biosynthetic genes [66,70]. Furthermore, the positive correlations between GNA and GBN (r = 0.428) and GNS (r = 0.339), as well as between PRO and GNS (r = 0.397), suggest that these GSLs may participate in similar plant defense mechanisms or metabolic pathways. The positive correlations between GTL and GBB (r = 0.489) and GBS (r = 0.329), and between GNS and GBB (r = 0.347) and GBS (r = 0.322), further support the idea of interconnected GSL metabolism within *Brassica* species [66,71]. The observed positive correlation between GBB and GBS (r = 0.308) reinforces the notion of a coordinated regulation or shared biosynthetic pathways among different GSLs. However, the negative correlations, such as those between GBN and SIN (r = −0.284) and between GER and GBN (r = −0.213), introduce a layer of complexity, suggesting that the increase in the levels of certain GSLs may be associated with the suppression of others, possibly due to competitive biosynthetic routes or differential regulation under various environmental or developmental conditions [66,72]. These findings underscore the intricate network of GSL metabolism in *Brassica* species, highlighting the potential to develop breeding and genetic engineering strategies aimed at enhancing desirable GSLs for improved plant defense and nutritional value [69]. Further research is needed to elucidate the underlying mechanisms of these correlations, which could lead to novel approaches to crop improvement [71,73]. 

### 3.4. GSLs In Silico Antioxidant Analysis

In silico docking analysis is a simulation technique used to identify the strong binding pose of a ligand with its target in the active site. This process entails selecting a 3D-coordinate space within the target’s binding site based on the binding affinity of the molecule, facilitating the formation of a complex. The GSLs present in *Brassica species* exhibit significant variability in occurrence among the analyzed accessions. To assess their pharmacological potential, we scrutinized them in terms of absorption, distribution, metabolism, and excretion using SwissADME [38]. This evaluation involved categorizing the compounds based on various physicochemical properties, including lipophilicity, water solubility, pharmacokinetics, drug likeness, and medicinal chemistry criteria. The GSL compounds listed are composed of 10–11 atoms and have TPSA values ranging from 199.79 (GBN, GNA, GNS, GTL and SIN) to 215.58 (GBS), 220.02 (PRO), 222.85 (GBB), 225.09 (GER) and 227.92 (GBE). The analyzed compounds also differed in terms of miLogP values, which affected their oral/intestinal absorption. The MW and MR of these compounds were less variable, reaching values ranging from 359.37 to 477.55 and from 76.23 to 103.43, respectively. This shows that GSLs have variable molecular properties. Among the compounds tested, the properties of GBN, GNA, GNS, GTL and SIN are more similar and non-inhibitory. GBN, GNA, GNS, GTL and SIN satisfied the rule of Lipinski with respect to being active drugs according to specific criteria such as a molecular weight (MW) of less than 500, a LogP value greater than 5, more than 10 hydrogen bond acceptors, and more than 5 hydrogen bond donors [74]. Furthermore, they satisfied Veber’s rules [75] and Ghose’s rules [76] by having 10 total hydrogen bonds, no more than 10 rotatable bonds, and a topological polar surface area (TPSA) of 199.79. In the drug-likeness analysis, we illustrated and tabulated all the compounds (Table S4, Figure S1) and predicted the biological activity using the Way2drug web server, which indicated high chemoprotective, anticancerous, and apoptosis activity (Table S5). 

**Figure 3:**
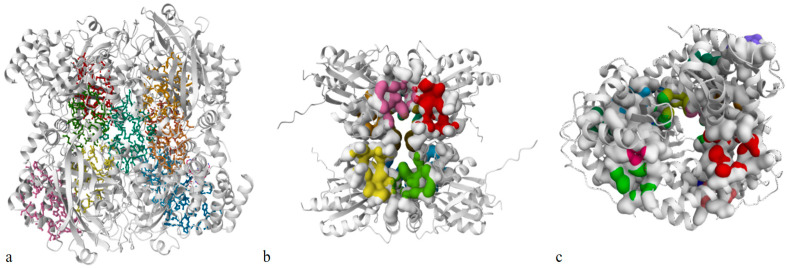
The binding sites coloured were predicted in the remodeled structure: (**a**) CAT, (**b**) GPX, and (**c**) SOD.

The selected targets of CAT, GPX and SOD, using PDB IDs 7VD9, 2I3Y and 7KKU, respectively, were re-modelled and assessed using the SWISS-MODEL Expasy workspace [42,43,44] (Figure S2, Table S6), and the model structures were visualized with a Ramachandran plot (Figure S3). The targets were subjected to a prediction of the binding sites of ligands for better interaction, revealing a high probability that there were more sites in CAT, while the numbers of sites in GPX and SOD were similar according to the results obtained using PrankWeb [45] (Figure 3, Table S7). The results show varying scores across parameters for the targets CAT, GPX, and SOD. Notably, CAT exhibited favorable Ramachandran values (96.27%), while GPX demonstrated the lowest Clash Score (0.33). However, SOD displayed the highest Ramachandran favored percentage (94.64%) and the most significant number of Rotamer outliers (8.33%). These findings highlight structural differences and quality variations among the proteins, offering insights into their potential biological functions. 

**Figure 4:**
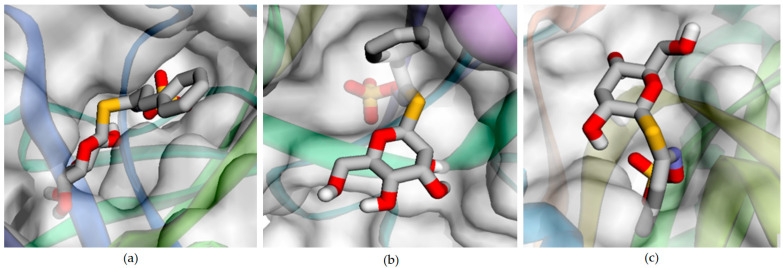
Results of molecular docking showing the binding sites of (**a**) catalase (CAT) with gluconastrutiin (GNS), (**b**) glutathione peroxide (GPX) with glucotropaeolin (GTL) and (**c**) superoxide dismutase (SOD) with sinigrin (SIN) complex structures.

Molecular docking analysis was performed using Webina [46], based on AutoDock Vina, which was run in online mode. The ligands GBN, GNA, GNS, GTL and SIN have varying affinities for the antioxidant enzymes CAT, GPX and SOD. The affinity values in kcal/mol indicate the strength of the interaction between the ligands and the enzymes and the RMSD values of the distance from the lower bound and best mode’s upper bound (Table 3). The CAT target, the ligand GNS, exhibited the highest affinity score of −8.580 kcal/mol, with a distance from the RMSD lower bound of 4.204 Å and a best-mode RMSD upper bound of 6.856 Å. Conversely, for the GPX target, the ligand GTL displayed the most favorable interaction, with an affinity score of 35.75 kcal/mol, along with a distance from the RMSD lower bound of 2.691 Å and a best-mode RMSD upper bound of 4.680 Å. Additionally, the ligand SIN demonstrated the strongest binding to the SOD target, with an affinity score of 20.040 kcal/mol, a distance from the RMSD lower bound of 2.320 Å, and a best-mode RMSD upper bound of 5.489 Å. These results warrant further analysis of drug activity via in vitro and in vivo studies. The AutoDock Vina-based Webina web tool provided complex affinity energy in both negative and positive values, indicating strong binding interactions between the targets and ligands. The interactions were visualized using the Webina tool [46], with the ligand binding the complex shown in the surface view, ligand hydrogen bonding shown in the dots view, and the ligands bound in the targets shown in the mesh view in Figure 4.

*Brassica* crops are recognized as superfoods containing phytochemicals with strong antioxidant properties with significant fungal inoculation effects that can increase GSL content, particularly in cabbage, kale and turnip greens [77]. Broccoli cultivars have glucobrassicin and glucoraphanin as principal components, with high levels of flavonoids and phenols contributing to their antioxidant activity [78]. Barillari et al. [79] identified glucoraphanin as a principal constituent of *Eruca sativa*, constituting approximately 95% of the total GSL content with an indirect antioxidant property. The ligands GBN, GNA, GNS, GTL and SIN interact with the antioxidant enzymes CAT, GPX and SOD with varying affinities, which are crucial for understanding their potential influence on oxidative-stress-related conditions. Oxidative stress is characterized by an imbalance between oxidants and antioxidants, leading to cellular damage [80]. Antioxidant enzymes like CAT, GPX and SOD play significant roles in mitigating oxidative stress by detoxifying harmful reactive oxygen species (ROS) [81]. The interaction of ligands with these enzymes can influence their activity, thus potentially modulating oxidative stress and providing therapeutic benefits in conditions such as cardiovascular diseases, neurodegenerative diseases, and cancer [82]. GNS, with the highest affinity for GPX, appears to possess a strong potential to influence this enzyme’s activity, which is involved in reducing lipid hydroperoxides and hydrogen peroxide [83]. Conversely, SIN’s lower affinity for CAT indicates a weaker potential to influence this enzyme’s role in hydrogen peroxide detoxification [84]. Specific affinities and interactions can guide the development of antioxidant remedies, which has been challenging due to the complexity of oxidative stress mechanisms and the limitations of antioxidant therapy [85]. These affinities are important for understanding the potential influence of these ligands on modulating the activity of antioxidant enzymes and managing oxidative-stress-related conditions [86]. CAT plays a role in hydrogen peroxide detoxification, GPX is involved in reducing lipid hydroperoxides and hydrogen peroxide and SOD is responsible for the dismutation of superoxide radicals [87,88,89,90,91]. The molecular interactions between ligands and antioxidant enzymes such as CAT, GPX, and SOD are crucial for understanding their potential therapeutic efficacy in combating oxidative-stress-related diseases. The affinity values, expressed in kcal/mol, indicate the strength of these interactions, with lower values suggesting stronger binding interactions. GBN exhibits the lowest affinity towards CAT at −7.09 kcal/mol, suggesting a weaker binding interaction with this enzyme compared to other ligands [92]. This could imply that GBN has less influence on antioxidative processes involving CAT. Conversely, GTL (35.75 kcal/mol) and GNS (38.21 kcal/mol) show high affinities towards GPX and SOD, indicating strong binding interactions. These interactions suggest a potential influence on the activity of scavenging reactive oxygen species (ROS) and oxidative stress and their therapeutic potential [93]. GNA displays the lowest affinity towards GPX among the ligands, with an affinity of −4.436 kcal/mol, showing weaker binding interactions and less influence on antioxidative processes involving GPX [94]. SIN (−6.90, 16.73 and 20.04 kcal/mol), with moderate affinities to all three targets, has moderate binding interactions and a weaker potential influence on antioxidative activity [95]. The interaction of ligands with antioxidant enzymes is a key factor in assessing their potential therapeutic efficacy. Ligands with higher affinity values towards these enzymes are likely to have stronger binding interactions, which could influence their effectiveness in scavenging ROS and mitigating oxidative stress [96].

Selective breeding represents a pivotal strategy in shaping the glucosinolate content of *Brassica* crops, aiming to enhance their nutritional and health-promoting properties. This approach involves manipulating the genetic makeup of *Brassica* crops to increase the concentration of specific GSLs, thereby maximizing their health-promoting effects. Among the *Brassica* accessions investigated, those of *B. rapa* subsp. *pekinensis* from the Taiwan landrace (TL), particularly with accessions 100352, 100353, and 100354, along with the Chinese cultivar (K037469), demonstrated the highest GSL content. Close behind were accessions of *B. rapa* subsp. *nipposinica* (TL-100394, 100406) and *B. rapa* subsp. *narinosa* from Chinese landrace (293390), Japan landrace (306666), and Taiwan landrace (100410). Conversely, *B. rapa* subsp. *rapa* from the Japan landrace (K037254) exhibited lower GSL levels. Interestingly, the Italian wild relative of *B. rapa* subsp. *campestris* displayed a high content of GNA and GBN but exhibited the lowest amount of GBB. This suggests a promising potential for use in breeding programs involving the Italian wild relatives and landraces of *B. rapa* subspecies to develop cultivars with elevated specific GSL content, thereby enhancing the health benefits for the human diet.

This breeding process entails the careful selection of parental lines based on their GSL profiles, followed by subsequent breeding to combine desirable traits [69]. Advanced molecular techniques, such as marker-assisted selection, can expedite the breeding process by enabling the selection of specific GSL traits [97]. Additionally, genomic approaches like quantitative trait loci (QTL) mapping can identify genetic loci associated with GNA and GBN content in *Brassica* crops [98]. This information can then be utilized to develop molecular markers for use in selective breeding programs aimed at enhancing the concentrations of these beneficial GSLs. Selective crop breeding holds significant promise for enhancing the GNA and GBN content of *Brassica* crops, thereby maximizing their nutritional and health-promoting properties.

## 4. Conclusions

This investigation underscores the remarkable potential of *Brassica* vegetables in elevating human health and combating diseases. With their abundant glucosinolates, these vegetables stand as potent sources of antioxidants. The diverse glucosinolate profiles across different *Brassica* species offer fertile ground for targeted breeding efforts aimed at optimizing antioxidant properties. The main contributors to the four principal components explaining 70.14% of the total variance were Sinigrin, Glucoberteroin, Glucoerucin and Glucotropaeolin content, revealing the significance of principal components in capturing variance within the glucosinolates. These results provide key insights for the selection and enhancement of *Brassica* varieties with superior glucosinolate profiles, poised to deliver valuable health benefits. Furthermore, our in silico molecular docking studies shed light on the intricate interactions between specific glucosinolates and antioxidant enzymes. By revealing the varying affinities of glucosinolates like Sinigrin and Gluconapin for enzymes such as catalase, glutathione peroxidase, and superoxide dismutase, we gained invaluable information for refining breeding strategies to maximize antioxidant potential. Our in silico study showed that glucobrassicanapin has a low affinity towards catalase at −7.09 kcal/mol, implying a weaker binding interaction with this enzyme and less influence in antioxidative processes involving catalase. On the other hand, glucotropaelin (35.75 kcal/mol) and gluconasturtiin (38.21 kcal/mol) showed high affinities towards glutathione peroxidase and superoxide dismutase, suggesting their strong binding interactions and, consequently, a potential influence on the activity of scavenging ROS and oxidative stress, as well as their therapeutic potential. Further studies are necessary to validate the significance of these ligand–target interactions observed through molecular docking simulations and to elucidate their biological effects accurately. Understanding these molecular interactions is crucial for the development of effective therapeutic agents targeting oxidative-stress-related diseases. The data suggest that ligands with lower affinities towards antioxidant enzymes may have a weaker influence on the target efficacy in scavenging ROS and mitigating oxidative stress, underscoring the importance of selecting ligands with optimal binding affinities for antioxidant targets in drug development. Our study contributes to the broader understanding of *Brassica* GSLs. By elucidating the mechanisms underlying their antioxidant effects, we advance the scientific foundation supporting the consumption of *Brassica* vegetables for attaining optimal health outcomes. This interdisciplinary synergy not only enriches our knowledge of *Brassica* metabolism but also offers tangible pathways for developing crops with enhanced nutritional and therapeutic value.

## Figures and Tables

**Table 1:** Coefficients and Eigenvalues of glucosinolate principal components.

	Coefficients of PC1	Coefficients of PC2	Coefficients of PC3	Coefficients of PC4
Glucobarbarin	0.39326	−0.03543	0.36776	−0.00975
Glucoberteroin	0.02899	0.67632	0.16737	0.03900
Glucobrassicanapin	0.49485	−0.11434	−0.14273	0.04016
Glucobrassicin	0.37983	0.00232	0.20934	0.20512
Glucoerucin	−0.09370	0.67273	0.08169	0.04366
Gluconapin	0.27930	0.04975	−0.40898	0.35562
Gluconasturtiin	0.44987	0.20539	−0.24152	0.18864
Glucotropaeolin	0.23415	−0.13393	0.60240	0.06097
Progoitrin	0.23710	0.07679	−0.40500	−0.48498
Sinigrin	−0.23486	−0.08482	−0.11558	0.74284
Eigenvalue	2.57277	1.84864	1.58271	1.00980
Variance %	25.73%	18.49%	15.83%	10.10%
Cumulative %	25.73%	44.21%	60.04%	70.14%

**Table 2:** Pearson co-efficient values of *Brassica* germplasm.

	**SIN**	**GNA**	**GBN**	**PRO**	**GTL**	**GER**	**GNS**	**GBE**	**GBB**
GNA	−0.042								
GBN	−0.284 **	0.428 **							
PRO	−0.177	0.140	0.209 *						
GTL	−0.134	−0.130	0.210 *	−0.179					
GER	−0.059	0.009	−0.213 *	−0.057	−0.103				
GNS	−0.027	0.339 **	0.518 **	0.397 **	−0.013	0.067			
GBE	−0.100	−0.024	−0.124	0.004	0.011	0.760 **	0.220 *		
GBB	−0.182	0.020	0.303 **	0.084	0.489 **	−0.115	0.347 **	0.078	
GBS	−0.165	0.148	0.329 **	0.036	0.304 **	−0.074	0.322 **	0.075	0.308 **

Note: **, correlation is significant at the 0.01 level (2-tailed); *, correlation is significant at the 0.05 level (2-tailed).

**Table 3:** Molecular docking of targets with selected glucosinolates (ligands) showing the scores of binding affinity and RMSD values.

**Targets**	**CAT**			**GPX**			**SOD**		
**Ligands**	**Affinity** **(kcal/mol)**	**Dist from rmsd l.b.**	**Best mode rmsd u.b.**	**Affinity** **(kcal/mol)**	**Dist from rmsd l.b.**	**Best mode rmsd u.b.**	**Affinity** **(kcal/mol)**	**Dist from rmsd l.b.**	**Best mode rmsd u.b.**
GBN	−7.090	2.749	5.965	−5.469	3.335	6.226	23.56	2.842	3.589
GNA	−6.814	2.769	4.162	−4.436	2.572	6.125	16.260	1.540	2.065
GNS	−8.580	4.204	6.856	31.42	0.886	1.55	38.210	2.468	5.772
GTL	−6.564	1.948	2.839	35.75	2.691	4.680	33.69	2.938	3.946
SIN	−6.902	2.756	5.709	16.73	2.651	3.516	20.040	2.320	5.489

Note: kcal/mol (Energy) is the predicted binding affinity. The calculation of RMSD values was based on the best mode, and only heavy atoms that can move were used. There are two types of RMSD (root mean square deviation) metrics, rmsd/lb and rmsd/ub, which differ in terms of how the distance is calculated between the atoms. (Dist.)—distance, (l.b.)—lower bound, and (u.b.)—upper bound.

## Data Availability

Data are contained within the article and Supplementary Materials.

## Supplementary Materials

The following supporting information can be downloaded at https://www.mdpi.com/article/10.3390/antiox13030376/s1. Figure S1: Egan graph illustrating the GSL structure with ADME properties of *Brassica* glucosinolates analyzed in SwissADME. Figure S2: Homology-modeled target graphs of three proteins: (a) catalase (CAT), (b) glutathione peroxidase (GPX), and (c) superoxide dismutase (SOD). Figure S3: The homology-modeled structures of three proteins: (a) catalase (CAT), (b) glutathione peroxidase (GPX), and (c) superoxide dismutase (SOD). Table S1: *Brassica* germplasm used for glucosinolate analysis. Table S2. *Brassica* glucosinolate quantity analysis. Table S3: GSL standards of the *Brassica* germplasm. Table S4: ADME properties of *Brassica* glucosinolates. Table S5: Biological activity of selected GSL compounds. Table S6: Homology-modelled target structure assessments. Table S7: Targets’ binding sites predicted for remodeled structures of CAT, GPX, and SOD.
