# Supplementary material for: Unveiling Glucosinolate Diversity in Brassica Germplasm and In Silico Analysis for Determining Optimal Antioxidant Potential
Source: Antioxidants (Basel). 2024 Mar 19;13(3):376. doi: 10.3390/antiox13030376 (PMC10968274; doi:10.3390/antiox13030376)
Supplement: Supplementary file 1 [file antioxidants-13-00376-s001.zip › antioxidants-2907666-supplementary.pdf]

Unveiling Glucosinolate Diversity in *Brassica* Germplasm and *In-silico* Analysis for Optimal Antioxidant Potential

Kanivalan Iwar [1, †], Kebede Taye Desta[1,2], Kingsley Ochar [1,3] and Seong-Hoon Kim [1, †,*]

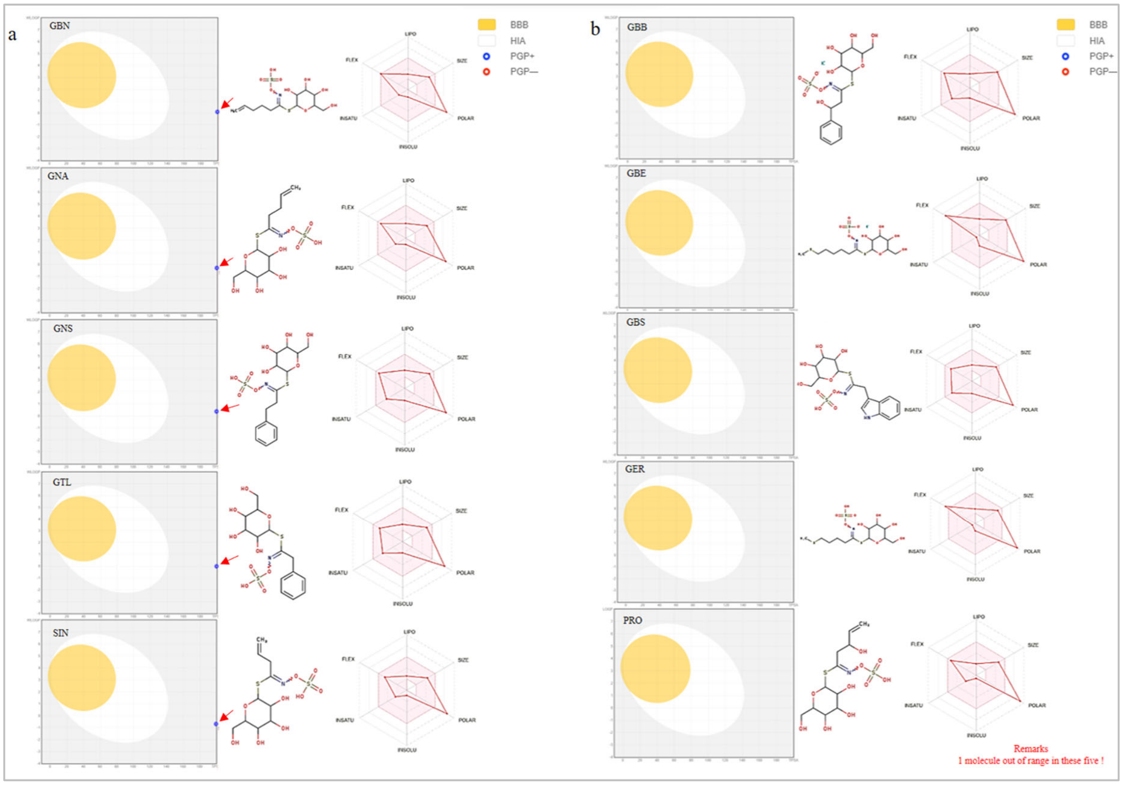

Figure S1. Egan graph illustrating the GSL structure with ADME properties of *Brassica* glucosinolates analyzed in SwissADME. a) GBN, GNA, GNS, GTL, and SIN are highlighted with PGP+ (arrow pointed), and b) GBB, GBE, GBS, GER, and PRO are noted with remarks indicating that one molecule is out of range in WLOGP and TPSA.

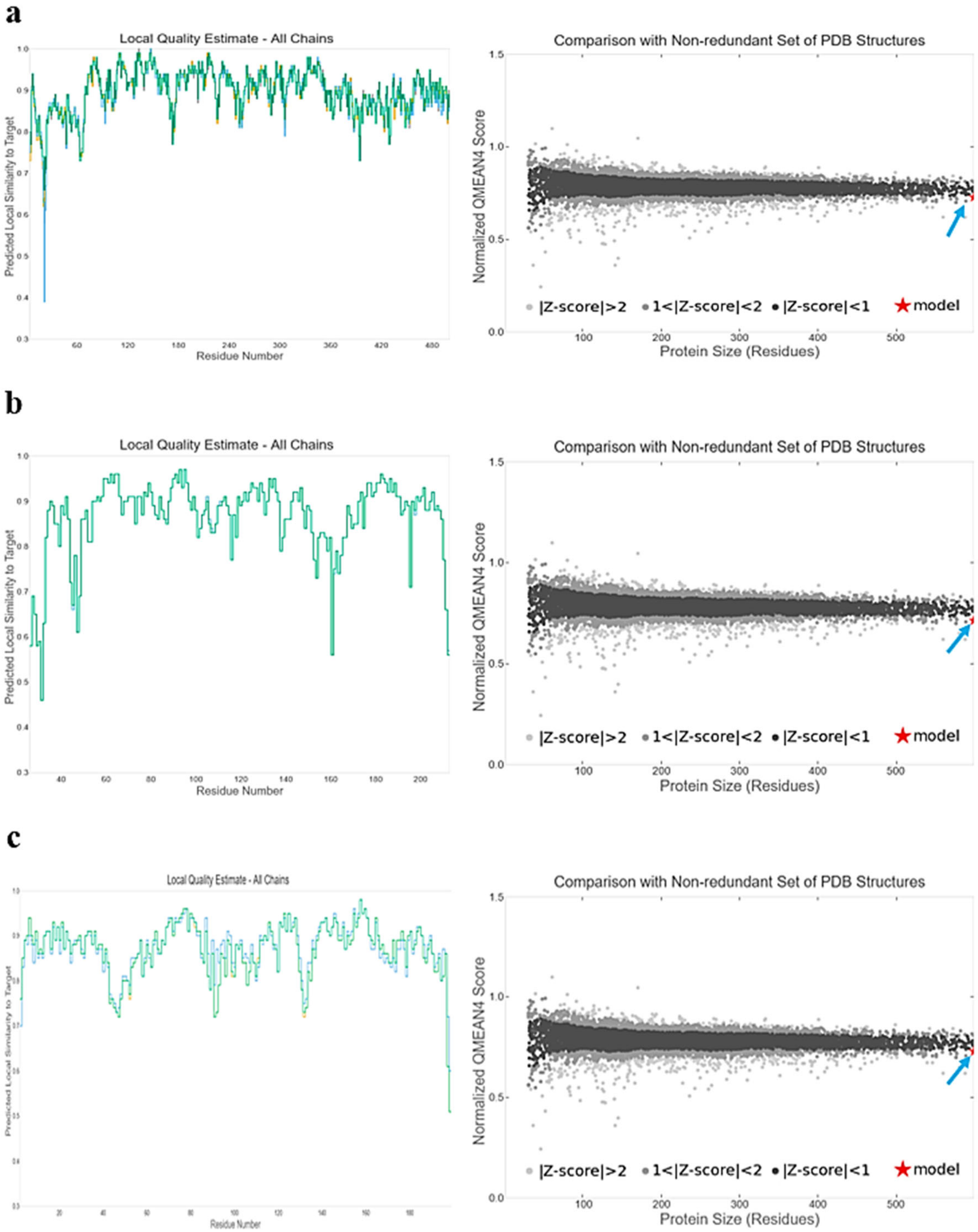

Figure S2. Homology-modeled target graphs of three proteins: a) Catalase (CAT), b) Glutathione peroxidase (GPX), and c) Superoxide dismutase (SOD). The figures include the QMEAN Disco and QMEAN graphs, with blue arrows indicating specific target points. Additionally, the protein residue dispersal is depicted row-wise for each target structure.

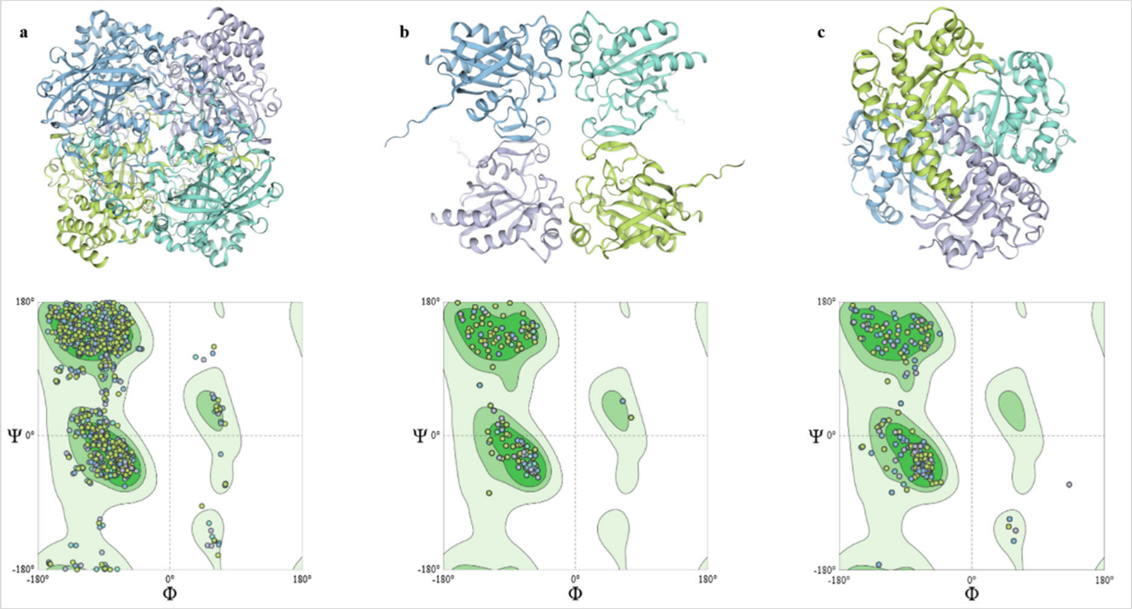

Figure S3. Displays the homology-modeled structures of three proteins: a) Catalase (CAT), b) Glutathione peroxidase (GPX), and c) Superoxide dismutase (SOD). These structures have undergone a Ramachandran Plot check using the SWISS-MODEL, ensuring the quality and reliability of the models

Table S1. *Brassica* germplasm used for Glucosinolates analysis.

Sl. No.	IT No.	*Brassica Species*	Crop name	Classifi-cation	Given Name	Origin
1.	339591	*Brassica oleracea* var. *medullosa* Thell.	Chou Moellier	Cultivar	U1	Uganda
2.	K000507	*Brassica rapa* subsp. *campestris* (L.) A.R.Clapham	Wild Turnip	Wild relative	8689	Italy
3.	100363	*Brassica rapa* subsp. *narinosa* (L.H.Bailey) Hanelt	Narinosa	Landrace	AVRDC-KJH-1985-100363	Taiwan
4.	100394	*Brassica rapa* subsp. *narinosa* (L.H.Bailey) Hanelt	Nipposinica	Landrace	AVRDC-KJH-1985-100394	Taiwan
5.	100410	*Brassica rapa* subsp. *narinosa* (L.H.Bailey) Hanelt	Narinosa	Landrace	AVRDC-KJH-1985-100410	Taiwan
6.	228170	*Brassica rapa* subsp. *narinosa* (L.H.Bailey) Hanelt	Narinosa	Landrace	99-2-9	Taiwan
7.	293079	*Brassica rapa* subsp. *narinosa* (L.H.Bailey) Hanelt	Narinosa	Landrace	TAH TSAI	Japan
8.	293390	*Brassica rapa* subsp. *narinosa* (L.H.Bailey) Hanelt	Narinosa	Landrace	Khe-ju-tatsai	China
9.	306666	*Brassica rapa* subsp. *narinosa* (L.H.Bailey) Hanelt	Narinosa	Landrace	110001 Chijimi Yukina	Japan
10.	100406	*Brassica rapa* subsp. *nipposinica* (L.H.Bailey) Kitam.	nipposinica	Landrace	AVRDC-KJH-1985-100406	Taiwan
11.	235637	*Brassica rapa* subsp. *nipposinica* (L.H.Bailey) Kitam.	Nipposinica	Cultivar	CGN07213	Japan
12.	235676	*Brassica rapa* subsp. *nipposinica* (L.H.Bailey) Kitam.	Nipposinica	Cultivar	040001 Sensuji Kyouna	Japan
13.	235677	*Brassica rapa* subsp. *nipposinica* (L.H.Bailey) Kitam.	Nipposinica	Cultivar	060002 Wase Mibuna	Japan
14.	32730	*Brassica rapa* subsp. *pekinensis* (Lour.) Kitam.	Chinese cabbage	Cultivar	Heonteun Baechu	Korea, South
15.	32731	*Brassica rapa* subsp. *pekinensis* (Lour.) Kitam.	Chinese cabbage	Cultivar	Shimoyama chitose	Japan
16.	32732	*Brassica rapa* subsp. *pekinensis* (Lour.) Kitam.	Chinese cabbage	Cultivar	Nozaki No. 2	Japan
17.	32733	*Brassica rapa* subsp. *pekinensis* (Lour.) Kitam.	Chinese cabbage	Cultivar	Song Dao Xin 2	China
18.	32735	*Brassica rapa* subsp. *pekinensis* (Lour.) Kitam.	Chinese cabbage	Cultivar	August meat	Korea, South
19.	32736	*Brassica rapa* subsp. *pekinensis* (Lour.) Kitam.	Chinese cabbage	Cultivar	Pyeonggangshin No. 1	Korea, South
20.	32737	*Brassica rapa* subsp. *pekinensis* (Lour.) Kitam.	Chinese cabbage	Cultivar	Large rhythm	Korea, South
21.	32739	*Brassica rapa* subsp. *pekinensis* (Lour.) Kitam.	Chinese cabbage	Landrace	Hotoren	China
22.	32740	*Brassica rapa* subsp. *pekinensis* (Lour.) Kitam.	Chinese cabbage	Landrace	Jia He	China
23.	32741	*Brassica rapa* subsp. *pekinensis* (Lour.) Kitam.	Chinese cabbage	Cultivar	Kyoto no.3	Japan
24.	32742	*Brassica rapa* subsp. *pekinensis* (Lour.) Kitam.	Chinese cabbage	Cultivar	Nozaki 2	Japan
25.	32744	*Brassica rapa* subsp. *pekinensis* (Lour.) Kitam.	Chinese cabbage	Cultivar	Harumakikyokuwase	Japan
26.	32747	*Brassica rapa* subsp. *pekinensis* (Lour.) Kitam.	Chinese cabbage	Landrace	Seoul	Korea, South
27.	32748	*Brassica rapa* subsp. *pekinensis* (Lour.) Kitam.	Chinese cabbage	Landrace	PI418957	China
28.	32749	*Brassica rapa* subsp. *pekinensis* (Lour.) Kitam.	Chinese cabbage	Landrace	PI418959	China

29.	32750	*Brassica rapa* subsp. *pekinensis* (Lour.) Kitam.	Chinese cabbage	Cultivar	Ching Pao 26	China
30.	32751	*Brassica rapa* subsp. *pekinensis* (Lour.) Kitam.	Chinese cabbage	landrace	Peking Hsiao Tsing Kou	China
31.	100330	*Brassica rapa* subsp. *pekinensis* (Lour.) Kitam.	Chinese cabbage	Cultivar	High-cold Summer	Korea, South
32.	100331	*Brassica rapa* subsp. *pekinensis* (Lour.) Kitam.	Chinese cabbage	Cultivar	Spring song	Korea, South
33.	100332	*Brassica rapa* subsp. *pekinensis* (Lour.) Kitam.	Chinese cabbage	Cultivar	Seventy-seven spoons	Korea, South
34.	100333	*Brassica rapa* subsp. *pekinensis* (Lour.) Kitam.	Chinese cabbage	Cultivar	Late Autumn 60 days	Korea, South
35.	100334	*Brassica rapa* subsp. *pekinensis* (Lour.) Kitam.	Chinese cabbage	Cultivar	Storage70days	Korea, South
36.	100335	*Brassica rapa* subsp. *pekinensis* (Lour.) Kitam.	Chinese cabbage	Cultivar	Sickness 60 days	Korea, South
37.	100336	*Brassica rapa* subsp. *pekinensis* (Lour.) Kitam.	Chinese cabbage	Cultivar	Struck	Korea, South
38.	100337	*Brassica rapa* subsp. *pekinensis* (Lour.) Kitam.	Chinese cabbage	Cultivar	Strong large rhythm	Korea, South
39.	100338	*Brassica rapa* subsp. *pekinensis* (Lour.) Kitam.	Chinese cabbage	Cultivar	Sammi Garak	Korea, South
40.	100339	*Brassica rapa* subsp. *pekinensis* (Lour.) Kitam.	Chinese cabbage	Cultivar	Garakshin No.1	Korea, South
41.	100340	*Brassica rapa* subsp. *pekinensis* (Lour.) Kitam.	Chinese cabbage	Cultivar	Long storge	Korea, South
42.	100341	*Brassica rapa* subsp. *pekinensis* (Lour.) Kitam.	Chinese cabbage	Cultivar	Josaeng Rhythm	Korea, South
43.	100342	*Brassica rapa* subsp. *pekinensis* (Lour.) Kitam.	Chinese cabbage	Cultivar	Inner Bottle Rhythm	Korea, South
44.	100343	*Brassica rapa* subsp. *pekinensis* (Lour.) Kitam.	Chinese cabbage	Cultivar	Ideal Rhythm	Korea, South
45.	100344	*Brassica rapa* subsp. *pekinensis* (Lour.) Kitam.	Chinese cabbage	Cultivar	Early Spring	Korea, South
46.	100345	*Brassica rapa* subsp. *pekinensis* (Lour.) Kitam.	Chinese cabbage	Landrace	Seoul cabbage	Korea, South
47.	100346	*Brassica rapa* subsp. *pekinensis* (Lour.) Kitam.	Chinese cabbage	Cultivar	Artificial cabbage	Korea, South
48.	100347	*Brassica rapa* subsp. *pekinensis* (Lour.) Kitam.	Chinese cabbage	Cultivar	Bamboo shoot cabbage	Korea, South
49.	100349	*Brassica rapa* subsp. *pekinensis* (Lour.) Kitam.	Chinese cabbage	Landrace	AVRDC-KJH-1985-100349	Taiwan
50.	100350	*Brassica rapa* subsp. *pekinensis* (Lour.) Kitam.	Chinese cabbage	Landrace	AVRDC-KJH-1985-100350	Taiwan
51.	100351	*Brassica rapa* subsp. *pekinensis* (Lour.) Kitam.	Chinese cabbage	Landrace	AVRDC-KJH-1985-100351	Taiwan
52.	100352	*Brassica rapa* subsp. *pekinensis* (Lour.) Kitam.	Chinese cabbage	Landrace	AVRDC-KJH-1985-100352	Taiwan
53.	100353	*Brassica rapa* subsp. *pekinensis* (Lour.) Kitam.	Chinese cabbage	Landrace	AVRDC-KJH-1985-100353	Taiwan
54.	100354	*Brassica rapa* subsp. *pekinensis* (Lour.) Kitam.	Chinese cabbage	Landrace	AVRDC-KJH-1985-100354	Taiwan
55.	100355	*Brassica rapa* subsp. *pekinensis* (Lour.) Kitam.	Chinese cabbage	Landrace	AVRDC-KJH-1985-100355	Taiwan
56.	100356	*Brassica rapa* subsp. *pekinensis* (Lour.) Kitam.	Chinese cabbage	Landrace	AVRDC-KJH-1985-100356	Taiwan
57.	100357	*Brassica rapa* subsp. *pekinensis* (Lour.) Kitam.	Chinese cabbage	Landrace	AVRDC-KJH-1985-100357	Taiwan
58.	100358	*Brassica rapa* subsp. *pekinensis* (Lour.) Kitam.	Chinese cabbage	Landrace	AVRDC-KJH-1985-100358	Taiwan

59.	100359	*Brassica rapa* subsp. *pekinensis* (Lour.) Kitam.	Chinese cabbage	Landrace	AVRDC-KJH-1985-100359	Taiwan
60.	K022386	*Brassica rapa* subsp. *pekinensis* (Lour.) Kitam.	Chinese cabbage	Cultivar	Kang bing 3 hao qiucai wang	China
61.	K023958	*Brassica rapa* subsp. *pekinensis* (Lour.) Kitam.	Chinese cabbage	Cultivar	Person heart	China
62.	K036310	*Brassica rapa* subsp. *pekinensis* (Lour.) Kitam.	Chinese cabbage	Landrace	DAK-SE	Korea, North
63.	K037469	*Brassica rapa* subsp. *pekinensis* (Lour.) Kitam.	Chinese cabbage	Cultivar	Re kang wang 58 tian	China
64.	K043728	*Brassica rapa* subsp. *pekinensis* (Lour.) Kitam.	Chinese cabbage	Landrace	Mostasa	Costa Rica
65.	K193859	*Brassica rapa* subsp. *pekinensis* (Lour.) Kitam.	Chinese cabbage	Cultivar	Wongyo No. 20037	Korea, South
66.	K243798	*Brassica rapa* subsp. *pekinensis* (Lour.) Kitam.	Chinese cabbage	Cultivar	KS 151	Thailand
67.	804344	*Brassica rapa* subsp. *rapa* L.	Turnip	Landrace	IncheonGanghwa1998-983406	Korea, South
68.	907305	*Brassica rapa* subsp. *rapa* L.	Turnip	Landrace	Moskovskij	Russia
69.	K002855	*Brassica rapa* subsp. *rapa* L.	Turnip	Landrace	MNG-KHH-2000-78	Mongolia
70.	K037254	*Brassica rapa* subsp. *rapa* L.	Turnip	Landrace	New Late Komatsuna	Japan
71.	K254291	*Brassica rapa* subsp. *rapa* L.	Turnip	Cultivar	Hua ye da man jing	China
72.	K255223	*Brassica rapa* subsp. *rapa* L.	Turnip	Landrace	SD 3255	Korea, South
73.	K255231	*Brassica rapa* subsp. *rapa* L.	Turnip	Landrace	SD 3271	Korea, South
74.	K255356	*Brassica rapa* subsp. *rapa* L.	Turnip	Landrace	SD 3431	Korea, South
75.	K257600	*Brassica rapa* subsp. *rapa* L.	Turnip	Landrace	GEO-PHJ-2016-15-19	Georgia
76.	119459	*Brassica sp.*	Brassica sp.	Landrace	PI311711	Chile
77.	216479	*Brassica sp.*	Brassica sp.	Cultivar	Heat-mustard	Unspecified
78.	216480	*Brassica sp.*	Brassica sp.	Cultivar	Old Mustard	Unspecified
79.	803359	*Brassica sp.*	Brassica sp.	Landrace	Mung choi	Unspecified
80.	K004273	*Brassica sp.*	Brassica sp.	Cultivar	Wirsing	Germany
81.	K018853	*Brassica sp.*	Brassica sp.	Landrace	WIR298	Unspecified
82.	K018856	*Brassica sp.*	Brassica sp.	Landrace	ASA DE CANTARO	Spain
83.	K226915	*Brassica sp.*	Brassica sp.	Landrace	Vishegorskayauluchshennaya	Vietnam
84.	K226916	*Brassica sp.*	Brassica sp.	Landrace	Sval of Victoria	Sweden
85.	K226918	*Brassica sp.*	Brassica sp.	Landrace	Nz Grandmaster	New Zealand
86.	K229552	*Brassica sp.*	Brassica sp.	Cultivar	PER-LYH-2013-1	Peru

87.	K229555	*Brassica sp.*	Brassica sp.	Cultivar	PER-LYH-2013-4	Peru
88.	K229557	*Brassica sp.*	Brassica sp.	Cultivar	PER-LYH-2013-6	Peru
89.	K229558	*Brassica sp.*	Brassica sp.	Cultivar	PER-LYH-2013-7	Peru

Table S2. *Brassica* Glucosinolates quantity analysis

Sample No.	IT No.	Scientific name	**SIN**	**GNA**	**GBN**	**PRO**	**GTL**	**GER**	**GNS**	**GBE**	**GBB**	**GBS**
1.	339591	*Brassica oleracea* var. *medullosa* Thell.	1648.65	98.60918	0.191017	159.3965	0.842697	2.520364	45.06257	0.493685	0	952.6867
2.	K000507	*Brassica rapa* subsp. *campestris* (L.) A.R.Clapham	6.018449	25027.2	5552.592	1370.853	9.054714	5.585607	228.2054	6.542671	2.25711	328.359
3.	100363	*Brassica rapa* subsp. *narinosa* (L.H.Bailey) Hanelt	10.06792	33049.23	3510.148	503.6124	9.140205	244.1044	417.1759	80.05511	51.41297	952.4496
4.	100394	*Brassica rapa* subsp. *narinosa* (L.H.Bailey) Hanelt	17.7065	13059.78	6027.519	1180.402	29.75818	65.42112	974.4683	344.3215	31.46682	1269.262
5.	100410	*Brassica rapa* subsp. *narinosa* (L.H.Bailey) Hanelt	1.803774	14800.57	3171.544	2256.432	10.5162	2888.747	715.6293	2287.308	10.95032	399.068
6.	228170	*Brassica rapa* subsp. *narinosa* (L.H.Bailey) Hanelt	9.612579	28780.31	2494.246	229.3948	3.591435	7.357058	290.3479	6.007654	33.25446	381.6054
7.	293079	*Brassica rapa* subsp. *narinosa* (L.H.Bailey) Hanelt	134.9702	26320.25	5928.834	349.9555	0.419313	1.088177	395.7977	0.269422	0	344.1843
8.	293390	*Brassica rapa* subsp. *narinosa* (L.H.Bailey) Hanelt	9.402096	12906.48	4848	2068.596	9.171145	6.077501	431.6096	39.17719	35.14676	475.3289
9.	306666	*Brassica rapa* subsp. *narinosa* (L.H.Bailey) Hanelt	8.003354	13086.28	6163.203	2047.693	5.66683	14.35192	622.6541	110.8664	10.32006	820.6429

10.	100406	*Brassica rapa* subsp. *nipposinica* (L.H.Bailey) Kitam.	0.394969	1790.217	343.3712	632.8642	6.224556	2203.825	560.7706	3217.829	33.14524	768.8473
11.	235637	*Brassica rapa* subsp. *nipposinica* (L.H.Bailey) Kitam.	6.171069	17545.24	2669.672	1662.31	2.901808	62.37011	431.1728	118.1737	3.030717	690.6473
12.	235676	*Brassica rapa* subsp. *nipposinica* (L.H.Bailey) Kitam.	12.72956	15199.2	6901.973	1170.514	5.534115	37.47964	1012.789	98.87868	56.00455	1210.792
13.	235677	*Brassica rapa* subsp. *nipposinica* (L.H.Bailey) Kitam.	15.16981	11681.84	5472.788	2682.207	9.906367	23.03045	1034.998	100.8037	70.19947	2098.265
14.	32730	*Brassica rapa* subsp. *pekinensis* (Lour.) Kitam.	0.379874	902.5799	2843.986	307.2265	25.69044	0.313958	236.2298	3.504784	76.8449	179.346
15.	32731	*Brassica rapa* subsp. *pekinensis* (Lour.) Kitam.	1.468344	1538.989	3639.522	711.3671	31.70249	0.671412	163.0862	2.726368	74.11225	72.89186
16.	32732	*Brassica rapa* subsp. *pekinensis* (Lour.) Kitam.	0.746331	2162.016	3525.455	376.0461	15.77512	1.649664	109.4789	10.15232	37.41373	695.0606
17.	32733	*Brassica rapa* subsp. *pekinensis* (Lour.) Kitam.	0.91153	1164.607	3090.866	551.3063	11.70168	1.013839	282.5336	8.977421	63.66022	229.8506
18.	32735	*Brassica rapa* subsp. *pekinensis* (Lour.) Kitam.	2.074633	632.7102	4301.165	640.7045	11.5828	0.445235	268.2527	2.267891	53.82328	268.44
19.	32736	*Brassica rapa* subsp. *pekinensis* (Lour.) Kitam.	2.818449	2230.411	8803.577	1082.342	21.57466	5.407671	773.649	168.2403	81.34092	600.1152
20.	32737	*Brassica rapa* subsp. *pekinensis* (Lour.) Kitam.	0.929979	914.8831	2952.501	940.3895	10.6937	3.943851	365.6183	43.35936	30.27076	236.492
21.	32739	*Brassica rapa* subsp. *pekinensis* (Lour.) Kitam.	1.515304	1913.69	2686.508	480.1995	17.49796	8.059312	168.4266	40.51206	32.81153	163.8662
22.	32740	*Brassica rapa* subsp. *pekinensis* (Lour.) Kitam.	0.524109	542.0345	711.6477	1867.709	14.15649	61.66153	323.1027	80.30769	28.30489	282.0342
23.	32741	*Brassica rapa* subsp. *pekinensis* (Lour.) Kitam.	0.537526	1100.804	954.6162	1332.673	11.70575	207.2867	476.3298	394.1707	18.86462	386.3322
24.	32742	*Brassica rapa* subsp. *pekinensis* (Lour.) Kitam.	8.277568	6815.379	4611.526	2294.977	16.12197	5.610123	598.9036	156.4516	38.03565	297.3014
25.	32744	*Brassica rapa* subsp. *pekinensis* (Lour.) Kitam.	3.242767	1905.886	6298.441	1021.716	13.69077	1.097667	477.0783	42.17834	59.64202	237.2486

26.	32747	*Brassica rapa* subsp. *pekinensis* (Lour.) Kitam.	1.093501	1393.914	2533.561	1837.94	4.190686	19.23764	190.9799	56.20742	59.22412	323.9866
27.	32748	*Brassica rapa* subsp. *pekinensis* (Lour.) Kitam.	0.721174	769.3912	2107	829.5557	14.64419	90.88414	551.2208	581.1183	49.69511	461.803
28.	32749	*Brassica rapa* subsp. *pekinensis* (Lour.) Kitam.	2.097275	3346.187	3760.559	1864.787	9.425175	80.21985	912.2416	186.5082	110.9534	723.3014
29.	32750	*Brassica rapa* subsp. *pekinensis* (Lour.) Kitam.	4.079665	2690.869	2742.606	3056.381	4.938935	126.0688	788.6108	562.9613	102.5612	1160.682
30.	32751	*Brassica rapa* subsp. *pekinensis* (Lour.) Kitam.	1.346751	2383.441	4299.402	986.3731	16.85149	4.260182	128.4699	31.88366	51.96739	1492.527
31.	100330	*Brassica rapa* subsp. *pekinensis* (Lour.) Kitam.	1.047379	1392.97	2423.142	1466.038	16.25468	64.1985	391.856	735.3555	79.22108	633.369
32.	100331	*Brassica rapa* subsp. *pekinensis* (Lour.) Kitam.	3.005451	1279.261	4587.398	1211.562	7.560658	7.67418	501.4136	170.0015	50.45355	1417.582
33.	100332	*Brassica rapa* subsp. *pekinensis* (Lour.) Kitam.	1.055765	901.589	2427.623	2103.374	23.3545	48.28865	499.6686	793.6923	61.09822	1017.491
34.	100333	*Brassica rapa* subsp. *pekinensis* (Lour.) Kitam.	2.045283	3413.014	4655.675	1291.729	21.92477	40.05694	300.8776	154.6445	57.98862	671.3363
35.	100334	*Brassica rapa* subsp. *pekinensis* (Lour.) Kitam.	0.434382	586.406	1538.44	1130.378	17.18613	4.953737	176.2755	29.17107	20.83276	609.0673
36.	100335	*Brassica rapa* subsp. *pekinensis* (Lour.) Kitam.	0.783229	2539.738	3250.222	817.4559	16.52825	16.76394	259.6497	85.28435	44.94956	686.5084
37.	100336	*Brassica rapa* subsp. *pekinensis* (Lour.) Kitam.	0.71195	2031.888	2199.599	855.7867	13.71845	14.88652	302.3699	46.89476	61.27114	678.188
38.	100337	*Brassica rapa* subsp. *pekinensis* (Lour.) Kitam.	0.651572	898.4208	1536.051	658.508	8.496173	11.09529	578.2157	29.94183	38.13728	338.1375
39.	100338	*Brassica rapa* subsp. *pekinensis* (Lour.) Kitam.	0.612159	774.8956	2089.985	1028.815	6.28725	3.285884	293.0516	64.71259	53.62837	337.7978
40.	100339	*Brassica rapa* subsp. *pekinensis* (Lour.) Kitam.	0.297694	620.5071	968.878	678.9454	5.519459	13.9913	320.0582	24.82817	17.3212	182.1665
41.	100340	*Brassica rapa* subsp. *pekinensis* (Lour.) Kitam.	0.612998	1432.984	1736.294	874.5592	7.736525	3.6552	251.9181	16.02832	19.17406	141.7094
42.	100341	*Brassica rapa* subsp. *pekinensis* (Lour.) Kitam.	0.463732	1149.304	1355.925	886.9885	6.528253	44.78766	577.0704	120.9713	21.25294	183.8811
43.	100342	*Brassica rapa* subsp. *pekinensis* (Lour.) Kitam.	1.241929	1580.51	4505.046	689.441	12.78293	0.468169	373.379	2.152315	67.38794	769.589

44.	100343	*Brassica rapa* subsp. *pekinensis* (Lour.) Kitam.	0.432704	1054.852	1932.658	756.5871	13.95538	1.925662	205.0382	15.65327	26.60523	479.4203
45.	100344	*Brassica rapa* subsp. *pekinensis* (Lour.) Kitam.	0.982809	2242.99	2058.56	1433.753	13.51979	15.92962	350.2991	88.71336	69.97118	232.9275
46.	100345	*Brassica rapa* subsp. *pekinensis* (Lour.) Kitam.	1.053249	951.664	1576.602	794.9024	5.357434	6.355081	279.5695	25.56831	11.57983	834.1888
47.	100346	*Brassica rapa* subsp. *pekinensis* (Lour.) Kitam.	5.584906	6292.443	7778.249	3187.047	17.26592	10.29735	696.4431	242.9238	47.37429	1681.803
48.	100347	*Brassica rapa* subsp. *pekinensis* (Lour.) Kitam.	1.875052	1999.402	4258.785	3377.309	7.607067	31.58244	856.5281	544.4432	109.008	328.5009
49.	100349	*Brassica rapa* subsp. *pekinensis* (Lour.) Kitam.	2.212159	3644.613	4673.914	941.6744	20.06106	16.65876	202.473	309.8362	17.74061	928.1442
50.	100350	*Brassica rapa* subsp. *pekinensis* (Lour.) Kitam.	3.687212	3917.649	5453.647	517.039	8.218531	4.744168	214.7139	72.9667	34.57869	683.1758
51.	100351	*Brassica rapa* subsp. *pekinensis* (Lour.) Kitam.	1.897694	1451.094	849.5035	1816.655	17.42062	36.10281	343.6718	50.23345	3.189989	481.2932
52.	100352	*Brassica rapa* subsp. *pekinensis* (Lour.) Kitam.	6.131656	9901.929	7422.057	3703.511	3.159095	52.66192	711.7418	479.9127	22.82139	194.8696
53.	100353	*Brassica rapa* subsp. *pekinensis* (Lour.) Kitam.	12.7153	14332.27	9803.821	3446.871	4.833903	41.40767	1007.876	208.1309	37.87107	497.6685
54.	100354	*Brassica rapa* subsp. *pekinensis* (Lour.) Kitam.	6.976939	12686.64	5182.537	8536.507	10.25566	127.6418	1258.672	627.814	150.6947	610.6436
55.	100355	*Brassica rapa* subsp. *pekinensis* (Lour.) Kitam.	7.370231	23501.58	6743.375	2388.358	6.555121	9.699486	1188.585	61.67088	93.59954	648.7521
56.	100356	*Brassica rapa* subsp. *pekinensis* (Lour.) Kitam.	3.490147	1175.522	5168.054	2991.745	40.77186	1.069197	736.3597	117.6096	53.64505	1134.857
57.	100357	*Brassica rapa* subsp. *pekinensis* (Lour.) Kitam.	0.698532	1433.896	1966.314	1636.766	16.25631	16.70779	271.5931	111.057	39.2992	250.7507
58.	100358	*Brassica rapa* subsp. *pekinensis* (Lour.) Kitam.	1.012159	1575.909	3312.058	1355.639	12.41492	5.699486	235.6372	74.66207	18.48237	800.524
59.	100359	*Brassica rapa* subsp. *pekinensis* (Lour.) Kitam.	1.745912	2741.934	5347.337	2128.235	11.57548	3.648873	234.3959	77.28971	45.88017	269.3898
60.	K022386	*Brassica rapa* subsp. *pekinensis* (Lour.) Kitam.	19.99832	3033.265	6991.591	4140.329	0.858981	19.67023	1058.173	193.2882	0	825.6945
61.	K023958	*Brassica rapa* subsp. *pekinensis* (Lour.) Kitam.	0.362264	726.4176	1081.162	179.6268	0.79873	0.264927	295.5624	4.427095	2.78157	473.6529

62.	K036310	*Brassica rapa* subsp. *pekinensis* (Lour.) Kitam.	9.553878	9845.84	6314.307	6736.526	0.552027	25.27244	1494.473	138.809	5.615472	1057.492
63.	K037469	*Brassica rapa* subsp. *pekinensis* (Lour.) Kitam.	14872.93	716.3373	0.228876	10.08903	4.944634	0.209569	385.5592	0.04822	5.953735	65.74062
64.	K043728	*Brassica rapa* subsp. *pekinensis* (Lour.) Kitam.	7.369392	5018.472	4286.92	231.439	1.465559	1.64571	228.4116	37.28052	0	112.4653
65.	K193859	*Brassica rapa* subsp. *pekinensis* (Lour.) Kitam.	1.314885	942.6852	992.4256	3748.225	0.919231	38.15184	576.4581	135.7497	3.258248	126.8272
66.	K243798	*Brassica rapa* subsp. *pekinensis* (Lour.) Kitam.	0.258281	51.75861	23.95113	406.8636	1.809966	728.2285	604.2794	607.3379	1.52901	255.5682
67.	804344	*Brassica rapa* subsp. *rapa* L.	1.080084	1309.468	5094.115	712.8822	1.350757	0.661131	579.7804	20.11251	4.241183	28.07432
68.	907305	*Brassica rapa* subsp. *rapa* L.	7.540461	2376.98	13.52349	9389.571	0.386745	137.8268	210.939	2.528129	0	340.987
69.	K002855	*Brassica rapa* subsp. *rapa* L.	10.15765	19649.24	5058.782	182.491	1.702491	18.57019	408.4069	52.13088	3.814562	201.9026
70.	K037254	*Brassica rapa* subsp. *rapa* L.	4.453669	1164.558	5061.402	2596.775	2.215437	3.246342	338.3613	108.1232	4.134243	477.6722
71.	K254291	*Brassica rapa* subsp. *rapa* L.	0.047799	159.7393	67.3576	718.9565	1.965478	505.9185	311.5238	456.7639	1.859689	219.3378
72.	K255223	*Brassica rapa* subsp. *rapa* L.	1.066667	748.4976	1241.307	2457.39	0.988438	21.73665	558.1826	138.6544	5.905954	253.1802
73.	K255231	*Brassica rapa* subsp. *rapa* L.	0.122432	195.1161	255.3941	839.4958	0.885035	498.087	404.7729	1176.204	2.998862	160.6845
74.	K255356	*Brassica rapa* subsp. *rapa* L.	0.090566	236.722	1.569437	282.1546	0.937144	2903.381	318.6919	602.4401	1.802806	67.36009
75.	K257600	*Brassica rapa* subsp. *rapa* L.	3.748428	3080.945	2510.028	7475.972	1.39391	230.6168	486.1165	186.421	71.63367	307.0858
76.	119459	*Brassica sp.*	11625.65	12640.75	48.49251	71.35764	3.804755	1.955714	750.6108	0.142365	5.828593	89.39279
77.	216479	*Brassica sp.*	12891.44	631.4605	11.21752	5.963876	3.196548	0.158165	320.8178	0	3.969662	72.7016
78.	216480	*Brassica sp.*	3.174004	7328.323	2729.139	739.2484	1.397981	53.22341	375.5317	109.318	2.52446	91.26273
79.	803359	*Brassica sp.*	3140.652	56.36851	0.081742	127.9815	0.950985	1.024911	54.15584	0	0.182025	1201.45
80.	K004273	*Brassica sp.*	806.2843	1.539904	0.035278	23.33761	0.33545	0.627916	10.6769	0	0.150171	131.6291
81.	K018853	*Brassica sp.*	54.47044	0.210677	0.074858	1.860983	0.304511	0.308422	3.478158	0	0.076223	350.5151

82.	K018856	*Brassica sp.*	1.567296	2490.535	1358.166	579.7552	0.832926	36.74891	291.2342	277.2147	2.321957	81.56076
83.	K226915	*Brassica sp.*	3.896017	701.1891	1215.139	4757.329	1.655268	66.40965	346.0063	162.5121	3.354949	190.8614
84.	K226916	*Brassica sp.*	3.16478	739.5153	531.0601	3017.661	3.99772	11.03124	282.4132	27.36625	2.885097	183.1133
85.	K226918	*Brassica sp.*	14.30021	3961.048	911.6262	7196.256	0.76779	8.062475	375.464	18.76311	3.476678	223.6871
86.	K229552	*Brassica sp.*	5.887631	4853.182	6874.516	2620.685	2.436085	0.797153	552.8264	26.06659	4.657565	156.5656
87.	K229555	*Brassica sp.*	5.071698	4113.902	8344.06	1958.178	1.250611	0.764729	520.6738	16.53349	4.279863	275.1089
88.	K229557	*Brassica sp.*	6.667505	9800.753	3640.294	6172.12	1.656896	8.375643	301.072	20.32223	2.657565	331.8774
89.	K229558	*Brassica sp.*	8.345493	15988.03	5456.923	12780.49	1.419964	19.29854	632.2676	28.02755	6.4562	260.9417

Table S3. GSLs standards of the *Brassica* Germplasm.

GSLs	Mean	Std Deviation	Sum	Mini	Median	Maxi	Sum of Squares	F value
Glucobarbarin	31.911	31.992	2840.122	0.027	22.821	150.694	6.520	945.291
Glucoberteroin	210.236	449.220	18711	0.028	74.662	3217.829	1.650	238705.4
Glucobrassicanapin	3256.895	2402.876	289863.7	0.035	2843.986	9803.821	2.970	4307049
Glucobrassicin	503.837	406.773	44841.540	28.074	340.987	2098.265	1.080	156286.2
Glucoerucin	136.834	491.601	12178.300	0.158	11.095	2903.381	1.280	185201.6
Gluconapin	5233.952	7244.347	465821.7	0.210	1913.690	33049.230	2.760	400007
Gluconasturtiin	456.980	289.426	40671.220	3.478	375.464	1494.473	6.060	87755.46
Glucotropaeolin	8.707	8.090	774.960	0.304	6.528	40.771	4.090	59.268
Progoitrin	1902.956	2235.534	169363.1	1.860	1130.378	12780.490	1.940	2806722
Sinigrin	511.179	2413.384	45494.970	0.047	2.212	14872.930	1.410	2052720

*GSLs- Glucosinolates, Std Dev.- Standard Deviation, Mini -Minimum, Maxi- Maximum; significance $p = 0$ ($p \geq 0.05$)

Table S4. ADME properties of *Brassica* glucosinolates.

Glucosinolates		GBB	GBE	GBN*	GBS	GER	GNA*	GNS*	GTL*	PRO	SIN*
Physicochemical properties	MW (<500 kD)	477.55	473.62	387.43	448.47	421.51	373.4	423.46	409.43	389.4	359.37
	H-ba (≤10)	11	10	10	10	10	10	10	10	11	10
	H-bd (≤5)	5	4	5	6	5	5	5	5	6	5
	MR	95.69	96.86	85.84	103.43	93.9	81.03	96.38	91.57	82.19	76.23
	TPSA (Å^2)	222.85	227.92	199.79	215.58	225.09	199.79	199.79	199.79	220.02	199.79
Lipophilicity	iLOGP	-3.05	-8.51	0.51	0.36	0.61	1.13	1.17	0.32	0.72	0.95
	XLOGP3	-1.03	-0.16	-0.41	-0.09	-0.51	-0.77	0.08	-0.22	-1.88	-1.07
	WLOGP	-0.81	0.31	0.09	0.46	0.27	-0.3	0.37	-0.02	-1.33	-0.69
	MLOGP	-2.15	-1.65	-2.01	-1.79	-1.92	-2.29	-1.39	-1.65	-3.06	-2.59
	Silicos-IT Log P	-2.1	-1.12	-1.65	-1.18	-1.5	-2.03	-1.35	-1.73	-2.76	-2.39
	Consensus Log P	-1.83	-2.22	-0.69	-0.45	-0.61	-0.85	-0.23	-0.66	-1.66	-1.16
Water solubility	ESOL Log S	-1.78	-1.95	-1.39	-2.33	-1.47	-1.14	-2.15	-1.95	-0.54	-0.93
	ESOL Class	V	V	V	V	V	V	S	V	V	V
	Ali Log S	-3.16	-4.17	-3.32	-3.98	-3.75	-2.95	-3.83	-3.52	-2.22	-2.64
	Ali Class	S	M	S	S	S	S	S	S	S	S
	Silicos-IT LogSw	0.46	0.33	1.15	-0.94	0.72	1.54	-0.48	-0.09	2.49	1.94
	Silicos-IT class	S	S	S	S	S	S	S	S	S	S
Pharmacokinetics	GI absorption	Low	Low	Low	Low	Low	Low	Low	Low	Low	Low
	BBB permeant	No	No	No	No	No	No	No	No	No	No

	P-gp substrate	No	Yes	Yes	No	Yes	Yes	Yes	Yes	Yes	Yes
	CYP inhibitors	0	0	0	0	0	0	0	0	0	0
	log Kp (cm/s)	-9.94	-9.3	-8.95	-9.1	-9.23	-9.12	-8.83	-8.95	-10.01	-9.25
Drug-likeness	LVs	1	0	0	2	0	0	0	0	2	0
	GVs	1	0	0	0	0	0	0	0	1	1
	VVs	1	2	1	1	1	1	1	1	1	1
	EVs	1	1	1	1	1	1	1	1	1	1
	MVs	2	1	1	2	1	1	1	1	3	1
	BS	0.55	0.55	0.11	0.11	0.11	0.11	0.11	0.11	0.11	0.11
Medicinal chemistry	PAINS alerts	0	0	0	0	0	0	0	0	0	0
	Brenk alerts	5	5	5	4	4	5	4	4	5	5
	LL	2	2	2	1	2	2	2	1	2	1
	SA (1-10)	5.53	5.81	5.53	5.16	5.72	5.5	5.42	5.27	5.63	5.35

Note: *Compound with positive ADME properties, MW- molecular weight, MR- molecular refractivity, TPSA- topological polar surface area, (H-ba)- Hydrogen bond acceptors, (H-bd)- Hydrogen bond donors, S- Soluble, Moderate soluble- M, Very soluble- V, CYP inhibitors - CYP1A2/CYP2C19/ CYP2C9/CYP2D6/ CYP3A4, LVs- Lipinski violations, GVs – Ghose Violations, VVs – Veber Violations, EVs – Egan Violations, MVs – Muegge Violations, BS- Bioavailability score, LL- Lead-likeness violations, SA- Synthetic accessibility.

Table S5. Biological activity of selected GSL compounds.

GBN		GNA		GNS		GTL		SIN	
Pa>0,7	Activity Predicted	Pa>0,7	Activity Predicted	Pa>0,7	Activity Predicted	Pa>0,7	Activity Predicted	Pa>0,7	Activity Predicted
0,975	Chemopreventive	0,964	Chemopreventive	0,956	Benzoate-CoA ligase inhibitor	0,959	Benzoate-CoA ligase inhibitor	0,964	Chemopreventive
0,956	Benzoate-CoA ligase inhibitor	0,956	Benzoate-CoA ligase inhibitor	0,917	Sugar-phosphatase inhibitor	0,945	Chemopreventive	0,960	Benzoate-CoA ligase inhibitor

0,927	Anticarcinogenic	0,927	Anticarcinogenic	0,909	Chemopreventive	0,922	Sugar-phosphatase inhibitor	0,939	Antineoplastic
0,913	Apoptosis agonist	0,924	Apoptosis agonist	0,869	Protein-Npi-phosphohistidine-sugar phosphotransferase inhibitor	0,901	Antineoplastic	0,916	Sugar-phosphatase inhibitor
0,903	Sugar-phosphatase inhibitor	0,893	Sugar-phosphatase inhibitor	0,866	Anticarcinogenic	0,884	Protein-Npi-phosphohistidine-sugar phosphotransferase inhibitor	0,906	Anticarcinogenic
0,887	Antineoplastic	0,878	Antineoplastic	0,866	Apoptosis agonist	0,828	Anticarcinogenic	0,908	Apoptosis agonist
0,869	Beta-adrenergic receptor kinase inhibitor	0,867	G-protein-coupled receptor kinase inhibitor	0,831	Beta-mannosidase inhibitor	0,819	Exoribonuclease II inhibitor	0,880	Protein-Npi-phosphohistidine-sugar phosphotransferase inhibitor
0,869	G-protein-coupled receptor kinase inhibitor	0,867	Beta-adrenergic receptor kinase inhibitor	0,810	Exoribonuclease II inhibitor	0,817	IgA-specific metalloendopeptidase inhibitor	0,872	G-protein-coupled receptor kinase inhibitor

* Pa- Probability of active

Table S6. Homology-modelled target structures assessments

Targets	CAT	GPX	SOD
MolProbity Score	1.15	0.91	1.95
Clash Score	0.51	0.33	1.45
Ramachandran Favoured	96.27%	98.92%	94.64%
Rotamer Outliers	1.98%	2.42%	8.33%
C-Beta Deviations	10	4	4

Cis Prolines	4/144	8/48	4/40
QMEANDisco Global	0.90±0.05	0.86±0.05	0.87±0.05
QMEAN	-0.82	-1.21	-0.95
Cβ	-0.32	-1.67	-0.96
All-atom	-0.86	0.16	0.28
Solvation	-1.44	-1.09	0.81
Torsion	-0.26	-0.59	-1.05
G-Factors	0.12	-0.03	0.23

Note: Ramachandra plot analysis with 118 structures of resolution of at least 2.0 Å with R-factor ≤ 20.0 would be a good model and expected above 90% score in the most favored regions. G-factors give an unusual (-0.5) and highly unusual (-1.0) measure values while below it.

Table S7. Target's binding site predicted for remodeled structure of CAT, GPX and SOD

Target	Pockets	Probability	Center_X	Center_Y	Center_Z	Residue_Ids
CAT	1	0.991	16.1178	39.2279	54.6048	B_112 B_114 B_131 B_133 B_146 B_147 B_148 B_153 B_158 B_161 B_216 B_217 B_218 B_299 B_332 B_334 B_350 B_353 B_354 B_357 B_358 B_361 B_362 B_365 B_72 B_73 B_74 B_75 C_61 C_65
	2	0.99	25.0904	7.9685	61.187	B_61 B_65 C_112 C_114 C_116 C_133 C_146 C_147 C_148 C_153 C_158 C_161 C_165 C_217 C_218 C_299 C_334 C_350 C_354 C_357 C_358 C_361 C_362 C_365 C_72 C_73 C_74 C_75
	3	0.99	7.8159	41.1275	84.8595	A_112 A_114 A_116 A_131 A_132 A_133 A_146 A_147 A_148 A_153 A_158 A_161 A_216 A_217 A_218 A_299 A_334 A_350 A_353 A_354 A_357 A_358 A_361 A_362 A_365 A_72 A_73 A_74 A_75 D_61 D_65
	4	0.989	30.9875	18.1448	90.0106	A_61 A_65 D_112 D_114 D_131 D_133 D_146 D_147 D_148 D_153 D_158 D_161 D_217 D_218 D_299 D_332 D_334 D_350 D_354 D_357 D_358 D_361 D_362 D_365 D_72 D_73 D_74 D_75
	5	0.879	19.2215	26.7619	72.5984	A_363 A_364 A_365 A_366 A_367 A_368 A_391 A_392 A_66 A_69 A_70 B_363 B_364 B_366 B_367 B_368 B_391 B_392 B_66 B_70 C_360 C_363 C_364 C_365 C_366 C_367 C_368 C_391 C_392 C_66 C_70 D_363 D_364 D_366 D_367 D_368 D_391 D_392 D_66 D_70
	6	0.83	40.7177	12.6888	106.581	D_149 D_151 D_152 D_194 D_198 D_201 D_203 D_212 D_213 D_215 D_233 D_235 D_237 D_242 D_282 D_302 D_303 D_304 D_305 D_442 D_445 D_446 D_450 D_451
	7	0.828	27.1335	-7.5347	49.5106	C_149 C_151 C_194 C_198 C_201 C_203 C_211 C_212 C_213 C_215 C_233 C_235 C_237 C_242 C_244 C_282 C_302 C_303 C_304 C_305 C_442 C_445 C_446 C_450 C_451 C_455
	8	0.81	14.2041	24.1783	90.1152	A_120 A_324 A_325 A_326 A_330 A_67 A_68 A_69 A_71 A_76 A_77 A_78 C_388 C_389 C_403 D_119 D_166 D_169 D_170 D_172 D_175 D_71

	9	0.81	13.2199	21.5111	56.2891	A_388 A_389 A_403 B_119 B_166 B_169 B_170 B_171 B_172 B_175 B_71 C_120 C_325 C_326 C_330 C_67 C_68 C_69 C_71 C_76 C_77 C_78
	10	0.797	24.4374	36.0115	88.0469	A_119 A_166 A_169 A_170 A_172 A_175 A_71 B_388 B_389 B_403 D_120 D_324 D_325 D_326 D_330 D_67 D_68 D_69 D_71 D_76 D_77 D_78
	11	0.773	-3.2192	52.0554	97.3278	A_151 A_152 A_194 A_198 A_203 A_213 A_215 A_235 A_237 A_242 A_282 A_302 A_303 A_304 A_305 A_310 A_442 A_445 A_446 A_450 A_451
	12	0.772	29.6372	23.8788	56.387	B_120 B_324 B_325 B_326 B_330 B_67 B_68 B_69 B_71 B_76 B_77 C_119 C_166 C_169 C_170 C_171 C_172 C_175 C_71 D_388 D_389 D_403
	13	0.709	39.141	25.4802	69.9303	B_30 B_59 B_63 D_363 D_368 D_369 D_371 D_372 D_377 D_378 D_381 D_383 D_385 D_387 D_388 D_394 D_395 D_396 D_397 D_398
	14	0.697	13.7156	50.0694	37.5749	B_149 B_151 B_194 B_198 B_203 B_213 B_215 B_233 B_235 B_237 B_242 B_282 B_302 B_304 B_305 B_442 B_445 B_446 B_450
	15	0.674	12.7438	26.5794	43.2329	B_117 B_118 B_119 B_121 B_122 B_123 B_126 B_127 B_128 B_168 B_170 B_177 B_178 B_179 B_182 B_186 B_199 B_200 B_254 B_466 C_121 C_123 C_254 C_255 C_258 C_259
	16	0.651	1.1766	26.5863	68.946	A_363 A_368 A_369 A_370 A_371 A_372 A_377 A_381 A_383 A_385 A_387 A_388 A_393 A_395 A_396 A_397 A_398 C_30 C_59 C_63
	17	0.641	11.355	10.7337	78.6126	A_30 A_59 A_63 C_363 C_368 C_370 C_371 C_372 C_377 C_378 C_383 C_385 C_387 C_389 C_393 C_394 C_395 C_397 C_398
	18	0.598	27.993	43.4802	73.7471	B_363 B_368 B_369 B_370 B_371 B_372 B_377 B_383 B_385 B_387 B_388 B_389 B_395 B_397 B_398 D_30 D_59 D_63
GPX	1	0.478	64.4875	10.1763	-7.5886	A_102 A_155 A_157 A_158 A_159 A_160 A_162 A_163 A_175 A_176 A_194 A_195 A_196 A_67 A_68 A_69 A_70
	2	0.478	40.0565	-10.1763	-7.5886	B_102 B_155 B_157 B_158 B_159 B_160 B_162 B_163 B_175 B_176 B_194 B_195 B_196 B_67 B_68 B_69 B_70
	3	0.478	64.4875	-10.1763	7.5886	C_102 C_155 C_157 C_158 C_159 C_160 C_162 C_163 C_175 C_176 C_194 C_195 C_196 C_67 C_68 C_69 C_70
	4	0.478	40.0565	10.1763	7.5886	D_102 D_155 D_157 D_158 D_159 D_160 D_162 D_163 D_175 D_176 D_194 D_195 D_196 D_67 D_68 D_69 D_70
	5	0.43	67.5096	2.0855	7.2433	A_101 A_104 A_106 A_132 A_133 A_134 A_165 A_97 A_98 C_100 C_101 C_102 C_118 C_67
	6	0.43	37.0344	-2.0855	7.2433	B_101 B_104 B_106 B_132 B_133 B_134 B_165 B_97 B_98 D_100 D_101 D_102 D_118 D_67
	7	0.43	67.5096	-2.0855	-7.2433	A_100 A_101 A_102 A_118 A_67 C_101 C_104 C_106 C_132 C_133 C_134 C_165 C_97 C_98
	8	0.43	37.0344	2.0855	-7.2433	B_100 B_101 B_102 B_118 B_67 D_101 D_104 D_106 D_132 D_133 D_134 D_165 D_97 D_98
SOD	1	0.878	17.8575	-7.4597	43.2137	A_162 A_30 A_31 A_34 A_35 A_38 A_67 A_68 B_117 B_118 B_119 B_121 B_163 B_166 B_172 B_174 B_175

2	0.878	-17.8575	7.4597	43.2137	C_162 C_30 C_31 C_34 C_35 C_38 C_67 C_68 D_117 D_118 D_119 D_121 D_163 D_166 D_172 D_174 D_175
3	0.877	18.8031	-1.0927	59.4645	A_117 A_118 A_119 A_120 A_121 A_161 A_163 A_166 A_172 A_174 B_162 B_30 B_31 B_34 B_35 B_38 B_67 B_68
4	0.877	-18.8031	1.0927	59.4645	C_117 C_118 C_119 C_120 C_121 C_161 C_163 C_166 C_172 C_174 D_162 D_30 D_31 D_34 D_35 D_38 D_67 D_68
5	0.076	9.7182	-10.8646	70.9393	B_3 B_72 B_73 B_76 D_53 D_55
6	0.076	-9.7182	10.8646	70.9393	B_53 B_55 D_3 D_72 D_73 D_76
